# Interventions for methamphetamine use among people on methadone maintenance treatment in Vietnam: a sequential multiple assignment randomized trial (STAR-OM)

**DOI:** 10.1016/j.lansea.2026.100773

**Published:** 2026-04-24

**Authors:** Le Minh Giang, Michael J. Li, Nguyen Thu Trang, Nguyen Bich Diep, Dao Thi Dieu Thuy, Dinh Thanh Thuy, Han Dinh Hoe, Hoang Thi Hai Van, Thai Thanh Truc, Hoa Hong Nguyen, Nguyen Ly Lai, Pham Thi Dan Linh, Vu Thi Tuong Vi, Cathy J. Reback, Arleen Leibowitz, Li Li, Chunqing Lin, Do Van Dung, Steven J. Shoptaw

**Affiliations:** a Centre for Training and Research on Substance Use & HIV, Hanoi Medical University, Hanoi, Vietnam; b School of Preventive Medicine and Public Health, Hanoi Medical University, Hanoi, Vietnam; c Center for Behavioral and Addiction Medicine, Department of Family Medicine, University of California, Los Angeles, Los Angeles, USA; d Faculty of Public Health, University of Medicine and Pharmacy at Ho Chi Minh City, Ho Chi Minh City, Vietnam; e South Vietnam HIV and Addiction Technology Transfer Center, University of Medicine and Pharmacy at Ho Chi Minh City, Ho Chi Minh City, Vietnam; f Friends Research Institute, Center for HIV Identification, Prevention and Treatment Services, University of California, Los Angeles, Los Angeles, CA, USA; g Department of Public Policy, Luskin School of Public Affairs, University of California, Los Angeles, Los Angeles, USA; h Department of Psychiatry and Biobehavioral Sciences, Jane & Terry Semel Institute for Neuroscience & Human Behavior, University of California, Los Angeles, Los Angeles, CA, USA

**Keywords:** Methamphetamine, Methadone, Contingency management, Opioids, Polysubstance use, Vietnam

## Abstract

**Background:**

Methamphetamine (MA) co-use with opioids has increased in Vietnam, hindering methadone maintenance therapy (MMT) through non-adherence and returning to opioid use. This study reports the primary outcomes of the “Screen, Treat and Retain people with opioid use disorders who use MA in methadone clinics” (STAR-OM) trial to integrate evidence-based behavioral interventions into MMT clinics and reduce MA co-use.

**Methods:**

STAR-OM was a sequential multiple assignment randomized trial (ClinicalTrials.gov Identifier: NCT04706624) with two 12-week intervention stages across 15 MMT clinics in Hanoi and Ho Chi Minh City, Vietnam. In the frontline intervention stage, 665 MMT patients who co-use MA were randomized to either a low-intensity frontline intervention of six weeks of contingency management (CM) followed by six weeks of group education, or a high-intensity frontline intervention of 12 weeks of CM. Participants who responded to treatment at the end of the frontline intervention stage—having 4 MA-negative urine drug screens (UDS) out of a possible 4 in Weeks 11 to 12—were reassigned to 12-weeks of theory-based text messaging for maintenance. Within each frontline intervention, those who did not have a treatment response were re-randomized to an adaptive intervention of either Matrix only or Matrix + CM for another 12 weeks. Segmented, two-level mixed effects logistic regression compared intervention effects on testing MA-negative over time.

**Findings:**

By Week 25, the low-intensity frontline intervention increased by 15.1% in the expected percentage of MA-negative UDS and the high-intensity frontline intervention increased by 16.9%. The high-intensity condition increased more rapidly in MA-negative UDS than the low intensity condition in the first 12 weeks. Participants who were reassigned to text messaging maintained a high percentage of MA-negative UDS—between 87.9% and 92.4%—from Week 14–25. Those re-randomized to Matrix + CM had a greater increase in MA-negative UDS than the Matrix only condition by 10.1 percentage-points.

**Interpretation:**

The STAR-OM trial provides a model of evidence-based adaptive interventions to reduce MA co-use in patients receiving MMT for opioid use disorder in Vietnam and globally. Consistent exposure to CM predicted greater reductions in MA use, emphasizing the importance of positive reinforcement in substance use intervention. Combining CM with the Matrix model may improve outcomes in those who do not initially respond to CM. Theory-based, unidirectional text messaging may support behavioral maintenance in people who respond to frontline intervention.

**Funding:**

This trial was funded by the National Institute of Drug Abuse and National Institute of Mental Health.

**Research in context**

**Evidence before this study**

We searched Google Scholar, PubMed, PsycINFO, and Cochrane databases through 2025 using terms including “methamphetamine,” “contingency management,” “Matrix model,” “text messaging,” and “methadone maintenance treatment.” Evidence from systematic reviews and randomized controlled trials indicates that contingency management is among the most effective behavioral interventions for reducing methamphetamine use, and the Matrix model has demonstrated superior reductions compared to non-standardized outpatient care. Theory-based text messaging has shown efficacy in reducing methamphetamine use and HIV-related risk behaviors. However, no trials were identified that had examined adaptive sequences of these interventions in methadone maintenance therapy settings.

**Added value of this study**

The STAR-OM trial is, to our knowledge, the among the first to use a sequential multiple assignment randomized trial design to evaluate adaptive behavioral interventions for reducing methamphetamine co-use in patients receiving methadone maintenance therapy. Conducted across 15 clinics in Vietnam, the trial demonstrated that 12 weeks of contingency management produced more rapid and sustained reductions than six weeks, that theory-based text messaging effectively maintained treatment gains in early responders, and that combining the Matrix model with contingency management produced greater reductions among those who did not initially respond to treatment.

**Implications of all the available evidence**

Combined with existing evidence, these findings suggest that adaptive sequences of evidence-based interventions can be successfully implemented in methadone maintenance therapy settings to address co-occurring methamphetamine use, with contingency management playing a central role at multiple treatment stages. This trial offers an example for addressing polysubstance use in low- and middle-income countries impacted by opioid-stimulant co-use. Future research should examine implementation in rural, primary care, and other substance use treatment settings.

## Introduction

The global rise of methamphetamine (MA) use in opioid-impacted areas has been linked to increased co-use. This has exacerbated risk of overdoses,^1^, ^2^, ^3^ chronic health conditions,^4^^,^^5^ HIV pathogenesis,^4^^,^^6^^,^^7^ non-adherence to opioid treatment programs, and returning to opioid use.^8^^,^^9^ Vietnam is experiencing an epidemic of MA co-use among patients receiving methadone maintenance therapy (MMT) for opioid use disorder (OUD),^10^^,^^11^ which has been associated with returning to heroin use and missed methadone doses.^8^^,^^11^^,^^12^ Research on integrating efficacious treatment strategies for co-occurring MA use in MMT clinics in Vietnam may provide a model for addressing polysubstance use in opioid treatment programs in other nations where opioid and MA use are comorbid, such as the United States^9^^,^^13^ and Canada.^14^

Currently, evidence-based behavioral interventions (EBIs) are the standard for MA use disorder,^15^ as there are no approved medications. This study examined four commonly used EBIs for MA use disorder: 1) **Motivational interviewing** is a person-centered, counseling approach to guide individuals in identifying goals, personal reasons for change, resolving ambivalence, and developing personalized strategies.^16^^,^^17^ Motivational interviewing delivered over either a single session or multiple sessions has shown efficacy for reducing substance use.^18^ 2) **Contingency management** (CM) is a positive reinforcement intervention that provides escalating-value vouchers or other reinforcers for consecutive negative urine drug screens (UDS),^19^^,^^20^ each reflecting multiple days of abstinence.^21^ Previous randomized controlled trials suggest CM has efficacy for reducing MA^19^^,^^20^ and other substance use,^22^ possibly greater than other evidence-based interventions up to one year of follow-up.^23^ CM may also be enhanced when paired with other psychosocial or behavioral interventions, such as cognitive behavioral therapy.^16^ 3) **The Matrix Model** shows superior reductions in MA use and related risk behaviors than non-standardized outpatient care.^16^^,^^24^ The Matrix model integrates different elements of cognitive and behavioral treatment approaches, including individual therapy, education on stimulant use consequences, craving management, relapse prevention training, and family education.^25^ The treatment procedures are manualized to ensure fidelity in new settings.^25^ 4) **Unidirectional, theory-based text messaging** has been shown to reduce MA use and HIV-related sexual risk behaviors^26^^,^^27^ and increase retention in HIV care,^28^ providing a cost-effective alternative to in-person therapies.^29^

Because no single intervention works for everyone, some individuals need additional or alternative approaches.^15^^,^^30^^,^^31^ Adaptive intervention strategies may optimize treatment response, providing extended support or augmented strategies for individuals who are slow to reduce or stop their MA use.^32^ The comorbid nature of MA use in MMT patients being treated for OUD underscores the need to integrate MA treatment into MMT clinics. This study delivered adaptive EBIs to patients with OUD who are in MMT to reduce their co-occurring MA use. The findings offer insights into the optimal sequencing and combination of EBIs to improve treatment outcomes for individuals using both opioids and MA.

## Methods

The “Screen, Treat and Retain people with opioid use disorders who use methamphetamine in methadone clinics” (STAR-OM) multisite trial^33^ (2020–2025) used a sequential multiple assignment randomized trial (SMART) design^32^ to evaluate interventions over two stages (NCT04706624). The SMART design embeds multiple randomizations within a single trial, enabling valid comparisons of interventions at each stage, joint effects of intervention sequences, and identification of optimal adaptive intervention strategies while maintaining internal validity at each decision point.^32^ A preceding pilot study with focus groups of MMT patients and providers (November 2020–February 2021) was published^34^ and informed this trial protocol.^33^ The present trial enrolled participants from 29 June 2021 to 1 April 2023, completing data collection 9 May 2024. Trained MMT providers conducted all trial activities across 15 MMT clinics in Vietnam, 8 in Hanoi and 7 in Ho Chi Minh City. A statistician uninvolved with enrollment block randomized participants 1:1 to one of two interventions at the start of the frontline intervention stage, balanced by sex and HIV status: 1) a low-intensity frontline intervention with six weeks of CM followed by six weeks of group education; or 2) a high-intensity frontline intervention of 12 weeks of CM.

Midpoint behavioral assessments were administered Week 13. Participants who met the criteria for a treatment response at the end of the frontline intervention stage—providing four out of a possible four MA-negative UDS in Weeks 11 to 12—were reassigned to receive theory-based text messaging during the adaptive intervention stage of the trial starting Week 14.

Those who did not have a treatment response in the frontline intervention stage were re-randomized 1:1 to receive enhanced treatment of either Matrix only or Matrix + CM in the adaptive stage, starting Week 14. Unlike the frontline stage, block randomization at this stage was by HIV status only. HIV representation required balancing for a forthcoming analysis to be published separately. This subsample, of which only 12.6% was female, was too small to include sex as a blocking factor. All adaptive interventions ended in Week 25, followed by an end-of-trial behavioral assessment in Week 26. The protocol of the STAR-OM trial has been previously published.^33^

**Fig. 1:**
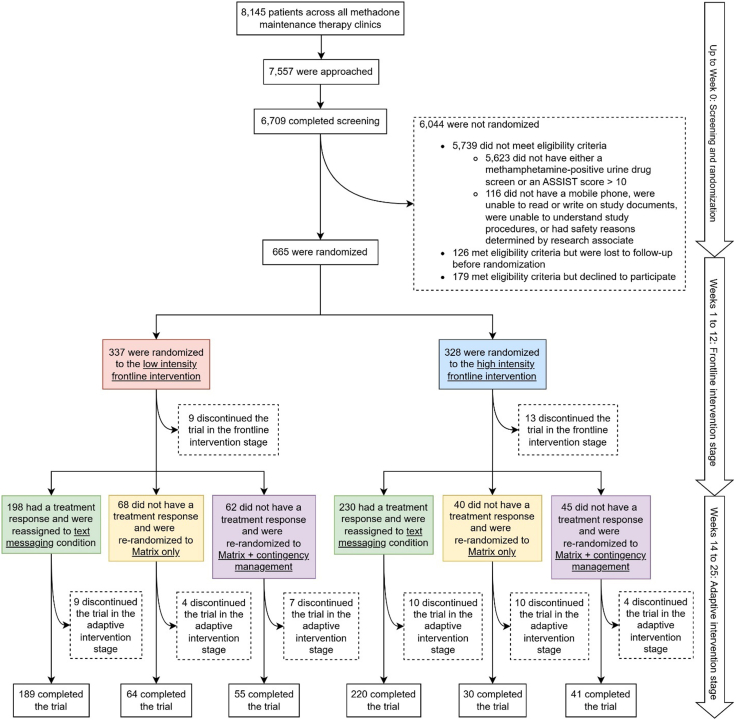
Trial design, screening, and randomization. Color coding indicates frontline intervention—low intensity (pink) and high intensity (blue)—and adaptive intervention assignment—text messaging (green), Matrix only (yellow), and Matrix + contingency management (purple). Note. All available observations from all randomized participants were analyzed under the intent-to-treat principle using maximum likelihood estimation.

Participants were patients receiving MMT for OUD who co-use MA in Vietnam. We recruited 665 patients across 15 MMT clinics, eight clinics in Hanoi and seven clinics in Ho Chi Minh City. Fig. 1 displays the numbers of individuals screened, randomized, re-randomized, and retained over the course of the trial. Participants were eligible if they: 1) were aged 16 years or older; 2) scored 10 or higher on the Alcohol, Smoking and Substance Involvement Screening Test (ASSIST)^35^ or confirmed MA use via UDS; 3) agreed to provide at least three forms of contact information; and 4) had a mobile phone that could receive text messages. Potential participants were ineligible if they: 1) had psychosis, other serious psychiatric conditions, or safety concerns or 2) were assessed as unable to understand study procedures.

Motivational interviewing was provided to all participants over two sessions—before the frontline intervention stage of the trial (Week 0) and midpoint of the trial (Week 13) between the frontline and adaptive intervention stages. The low-intensity frontline intervention condition offered six weeks of CM with escalating voucher reinforcement for consecutive MA-negative urine samples, up to $40 USD total value in voucher reinforcement. Testing MA-positive reset the reinforcer levels to the base value. After the six weeks of CM, participants received group education for another six weeks on: 1) addiction mechanism, 2) road to recovery, 3) coping with triggers, 4) boredom, 5) building trust, and 6) relapse prevention. The high-intensity frontline intervention provided 12 weeks of CM with up to $150 USD total value in vouchers, comparable to half the average monthly income^36^ and approximately three times the monthly cost of methadone treatment. This amount was calibrated to the local cost of living^37^ and informed by pilot findings and focus group discussions with MMT patients and providers in Vietnam from our formative research,^34^ with sustainability of implementation in Vietnamese MMT clinics as a key consideration.

The text messaging intervention delivered twice daily unidirectional scripted text messages for another 12 weeks, and was adapted from previous text messaging interventions by the co-authors^26^, ^27^, ^28^ based on Social Support Theory,^38^ Social Cognitive Theory,^39^ and the Health Belief Model.^40^

The Matrix-only condition retained the same core content and structure of the original Matrix model^25^ but was shortened from 24 to 12 weekly counseling sessions to fit the trial timeline. The original sequence was preserved to maintain internal logic. Content deemed not culturally suited to Vietnam (the 12-step program) or infeasible for the trial design (family education) were omitted.^34^ The Matrix + CM condition received both the weekly Matrix sessions and CM during the 12-week adaptive intervention stage with the same reinforcer structure as in the frontline intervention stage (i.e., up to $150 equivalent).

Urine drug screens (UDS) were conducted at baseline and twice weekly on randomly selected days for 25 weeks to evaluate treatment response and longitudinal changes in MA use over time. Treatment response to the frontline intervention was defined as testing MA-negative four out of a possible four times during Weeks 11–12, the endpoint of the frontline intervention stage. Not having a treatment response was defined as testing MA-positive at least once during that period. Treatment response to the adaptive intervention had the same criteria but during Weeks 24–25, the endpoint of the adaptive intervention stage.

At baseline, Week 13, and Week 26 assessment visits, participants separately self-reported their number of days using two types of MA in the past 30 days—crystal MA and tablet MA. To create an index of days of any MA use in the past 30 days, we took the maximum of the two values rather than summing them, which would have produced values exceeding 30 days and been uninterpretable as a proportion of a 30-day period. Self-reported MA use exhibited substantial zero-inflation (i.e., a large proportion reporting no past-30-day use) and overdispersion (variance greatly exceeding the mean) too extreme for zero-inflated count models, which failed to converge. Therefore, self-reported MA use was re-coded into 0 days, 1–5 days, or 6 or more days of use in the past 30 days.

Adverse events were systematically assessed and recorded at each study visit through structured discussions with participants and providers by trained research staff.

### Statistical analysis

The trial was powered to detect differences of 20% or more in binary substance use outcomes between frontline intervention groups, assuming base rates of 80–90%, 80% power, a 5% alpha level, and 20% attrition, yielding a target sample of at least 600 participants.^33^

The primary outcome was providing a MA-negative UDS, assessed twice weekly. Analyses used segmented, two-level mixed effects logistic regression with the Stata command “melogit” to examine the relationship between intervention assignment and testing MA-negative over time.^41^ Given high overall retention (90%), models for each stage were estimated via maximum likelihood under the intent-to-treat principle, including all participants randomized or reassigned at each stage and retaining all available observations regardless of dropout.^42^ Models included random intercepts for unique MMT clinic and participant identifiers to adjust for within-clinic and within-person associations. Neither HIV status, sex, nor other socio-demographic measures were associated with intervention assignment, indicating successful randomization. Akaike's and Schwarz's Bayesian information criteria, computed using the Stata command ‘estat ic,’ indicated better model fit—lower values—without these factors,^43^ which were thus excluded. For ease of interpretation, predicted probabilities of testing MA-negative were estimated using the Stata command “margins,” then reported and plotted as expected percentages of MA-negative UDS.^44^

To estimate change in odds of testing MA-negative between low-intensity and high-intensity frontline interventions, predictors included frontline intervention, time, and time-by-frontline intervention interaction. Time was modeled in segments with breakpoints at Weeks 6, 12, and 18 to capture slope changes in log odds of MA-negative UDS.^45^ Week 6 marked the end of CM, Week 12 the end of the frontline phase, and Week 18 an observed slope change identified using locally weighted scatterplot smoothing (LOWESS).^46^

$$\text{logit}\left(P\left(Y_{tij}=1\right)\right)=\beta_{0}+\beta_{1}{\text{FRT}}_{i}+\sum_{k=1}^{3}\beta_{2k}t_{kij}+\sum_{k=1}^{3}\beta_{3k}\left({\text{FRT}}_{i}\times t_{kij}\right)+u_{j}+u_{ij}$$

where Y_t_ᵢⱼ = MA-negative UDS (binary) for timepoint t, participant i, clinic j; FRTᵢ = frontline intervention (0 = low-intensity, 1 = high-intensity); β_0_ = intercept; β_1_ = main effect of frontline intervention; t_k_ = time segments (k = 1, 2, 3) with breakpoints at Weeks 6, 12, and 18; β_2k_ = slope for each time segment; β_3k_ = time-by-intervention interaction for each segment; uⱼ = random intercept for clinic j; uᵢⱼ = random intercept for participant i within clinic j.

To compare Matrix only and Matrix + CM adaptive interventions on testing MA-negative, predictors included adaptive intervention assignment, time, and their interaction, with breakpoints in time at Week 14 (start of the adaptive intervention) and Week 18 identified with LOWESS.

$$\text{logit}\left(P\left(Y_{tij}=1\right)\right)=\beta_{0}+\beta_{1}{\text{ADP}}_{i}+\sum_{k=1}^{2}\beta_{2k}t_{kij}+\sum_{k=1}^{2}\beta_{3k}\left({\text{ADP}}_{i}\times t_{kij}\right)+u_{j}+u_{ij}$$

where ADPᵢ = adaptive intervention assignment (0 = Matrix only, 1 = Matrix + CM); t_k_ = time segments (k = 1, 2) with breakpoints at Weeks 14 and 18. All other terms as defined in the frontline intervention model above.

The joint effects of frontline and subsequent adaptive interventions by time were modeled as a three-way interaction with the same time breakpoints to examine whether adaptive intervention outcomes differed by prior frontline intervention assignment.

$$\text{logit}\left(P\left(Y_{tij}=1\right)\right)=\beta_{0}+\beta_{1}{\text{FRT}}_{i}+\beta_{2}{\text{ADP}}_{i}+\beta_{3}\left({\text{FRT}}_{i}\times {\text{ADP}}_{i}\right)+\sum_{k=1}^{3}\beta_{4k}t_{kij}+\sum_{k=1}^{3}\beta_{5k}\left({\text{FRT}}_{i}\times t_{kij}\right)+\sum_{k=1}^{3}\beta_{6k}\left({\text{ADP}}_{i}\times t_{kij}\right)+\sum_{k=1}^{3}\beta_{7k}\left({\text{FRT}}_{i}\times {\text{ADP}}_{i}\times t_{kij}\right)+u_{j}+u_{ij}$$

where β_3_(FRTᵢ × ADPᵢ) = frontline intervention-by-adaptive intervention interaction; t_k_ = time segments (k = 1, 2, 3) beginning Week 14 with breakpoints at Weeks 18 and 25; β_4k_ = slope for each time segment; β_5k_ = frontline-by-time interaction for each segment; β_6k_ = adaptive-by-time interaction for each segment; β_7k_ = three-way frontline-by-adaptive-by-time interaction for each segment. All other terms as defined in the frontline and adaptive models above.

Text messaging participants were modeled as both one group and stratified by prior frontline intervention, with no time breakpoints since LOWESS showed no slope changes. For the comparison by prior frontline intervention among those receiving text messaging, frontline intervention and a time-by-frontline intervention interaction term were added as covariates.

Separate mixed logistic regression models evaluated treatment response (defined above) at the end of the frontline stage (Weeks 11–12) and end of trial (Weeks 24–25), comparing low-vs. high-intensity frontline interventions; Matrix only vs. Matrix + CM; text messaging as one combined group; and text messaging stratified by prior frontline intervention. All models included a random intercept for MMT clinic, and marginal probabilities were obtained for each condition.

Separate two-level ordered logistic regressions evaluated changes in self-reported MA use by frontline intervention and by adaptive intervention, with random intercepts for clinic and participant, and predictors including intervention, discrete time (baseline for frontline comparisons only, Week 13, and Week 26), and their interaction. Predicted probabilities were computed from these models and reported as expected percentages of using MA on 0 days, 1–5 days, and 6–30 days in the past 30 days over time.

Sensitivity and specificity of self-reported MA use in the past 30 days were calculated against UDS results from the previous 4 weeks. Sensitivity was the percentage of participants reporting any use in the past 30 days among those who tested positive at any visit during Weeks 9–12; specificity was the percentage reporting no use among those who tested negative at all visits.

The trial registration (ClinicalTrials.gov, NCT04706624) lists reduced MA use, viral suppression, and reduced HIV sexual risk behaviors across the frontline and adaptive stages as primary outcomes, and heroin-negative UDS, opioid overdose frequency, antiretroviral therapy adherence, HIV testing frequency, and quality of life as secondary outcomes. Given the volume of analyses for each domain and that the STAR-OM interventions directly address MA use, the planned HIV primary outcomes and secondary outcomes will be reported separately in forthcoming publications. Furthermore, the results presented here differ from those reported on ClinicalTrials.gov, which requires reporting MA abstinence in raw counts and unadjusted percentages; the expected percentages reported here were instead marginalized from mixed-effects models used to account for the multisite, longitudinal design.

### Ethics statement

The STAR-OM trial was registered on ClinicalTrials.gov (NCT04706624) on 13 January 2021 and approved by the Ethics Committee of Hanoi Medical University on 8 June 2020. Annual reports and all serious adverse events were submitted to the Ethics Committee. The Data and Safety Monitoring Board for Addiction Medicine of the University of California, Los Angeles independently reviewed trial data twice yearly and provided written recommendations to the principal investigators. An independent scientific advisory committee met annually to review and advise on scientific merit, rigor, and key findings.

Given participants' vulnerability to legal or social repercussions in Vietnam, the study obtained formal approval for participant safeguards from the Vietnam Ministry of Health. Written informed consent was obtained in a private setting by trained staff experienced in serving people who use substances, explaining voluntariness, confidentiality, and the right to withdraw at any time without impacting their care. Signed consent forms were stored and locked separately from research data. Clinics retained full authority over urine test confidentiality, all study staff signed confidentiality agreements, and participants were identified by unique codes. Participants received modest remuneration (approximately $10 USD per visit) for their time, effort, travel, and opportunity costs for attending comprehensive baseline, Week 13, and Week 26 assessment visits.^47^ CM intervention vouchers were redeemable only for methadone treatment fees or personal goods (e.g., shampoo, body wash, tea, coffee, children's toys).

### Role of the funding source

This trial was funded by the National Institute on Drug Abuse (R01DA050486; PIs: Giang and Shoptaw) of the U.S. National Institutes of Health. The funder had no role in study design, data collection, data analysis, interpretation of findings, or dissemination.

## Results

### Baseline characteristics

Table 1 summarizes baseline participant characteristics stratified by frontline and adaptive intervention. The mean age was 41 years. About 89% were male, 43% were married, 70% had less than a 12th grade education, and 63% had a paid job. About 54% attended MMT clinics in Hanoi and the remainder in Ho Chi Minh City. About 20% were living with HIV, 62% tested positive for MA, and the mean ASSIST score was 16.5 (moderate risk is 4–26).^35^ These characteristics did not significantly differ by frontline intervention assignment.

**Table 1:** Baseline participant characteristics by frontline intervention and adaptive intervention assignment.

	Total (n = 665)	Frontline intervention (Weeks 1–12)		Adaptive Intervention (Weeks 14–25)		
		Low intensity (n = 337)	High intensity (n = 328)	Text messaging (n = 428)	Matrix only (n = 108)	Matrix + CM (n = 107)
	M (SD)	M (SD)	M (SD)	M (SD)	M (SD)	M (SD)
Age	41.4 (7.4)	41.5 (7.5)	41.4 (7.2)	42.2 (7.5)	40.0 (6.7)	40.2 (7.2)
ASSIST score	16.5 (8.2)	16.7 (8.3)	16.3 (8.1)	16.1 (8.0)	17.1 (8.8)	17.7 (8.5)
	n (%)	n (%)	n (%)	n (%)	n (%)	n (%)

Sex assigned at birth						
Male	591 (88.9)	298 (88.4)	293 (89.3)	384 (89.7)	96 (88.9)	92 (86.0)
Female	74 (11.1)	39 (11.6)	35 (10.7)	44 (10.3)	12 (11.1)	15 (14.0)
HIV status						
Negative	533 (80.2)	262 (77.7)	271 (82.6)	343 (80.1)	87 (80.6)	86 (80.4)
Positive	132 (19.9)	75 (22.3)	57 (17.4)	85 (19.9)	21 (19.4)	21 (19.6)
Currently married						
No	377 (56.7)	198 (58.8)	179 (54.6)	223 (52.1)	64 (59.3)	77 (72.0)
Yes	288 (43.3)	139 (41.2)	149 (45.4)	205 (47.9)	44 (40.7)	30 (28.0)
Education						
Less than 12th grade	467 (70.2)	242 (71.8)	225 (68.6)	290 (67.8)	82 (75.9)	77 (72.0)
12th grade or higher	198 (29.8)	95 (28.2)	103 (31.4)	138 (32.2)	26 (24.1)	30 (28.0)
Has a paid job						
No	249 (37.4)	124 (36.8)	125 (38.1)	156 (36.4)	42 (38.9)	42 (39.3)
Yes	416 (62.6)	213 (63.2)	203 (61.9)	272 (63.6)	66 (61.1)	65 (60.7)
Region						
Hanoi	358 (53.8)	181 (53.7)	177 (54.0)	291 (68.0)	33 (30.6)	28 (26.2)
Ho Chi Minh City	307 (46.2)	156 (46.3)	151 (46.0)	137 (32.0)	75 (69.4)	79 (73.8)
Methamphetamine urine drug screen						
Negative	252 (37.9)	122 (36.2)	130 (39.6)	–	–	–
Positive	413 (62.1)	215 (63.8)	198 (60.4)	–	–	–

Notes. M = mean; SD = standard deviation; n = count; % = column percentage.

Fig. 1 illustrates the counts for screening, randomization, and retention over the trial period. Retention was 96.7% (643 out of 665) at the end of the frontline stage and 93.2% (599 out of 643) in the adaptive stage—defined as providing at least one of four urine samples in Weeks 24–25 and completing the Week 26 assessment—yielding 90.1% (599/665) overall.

### Primary outcome

#### Frontline intervention effects

**Fig. 2:**
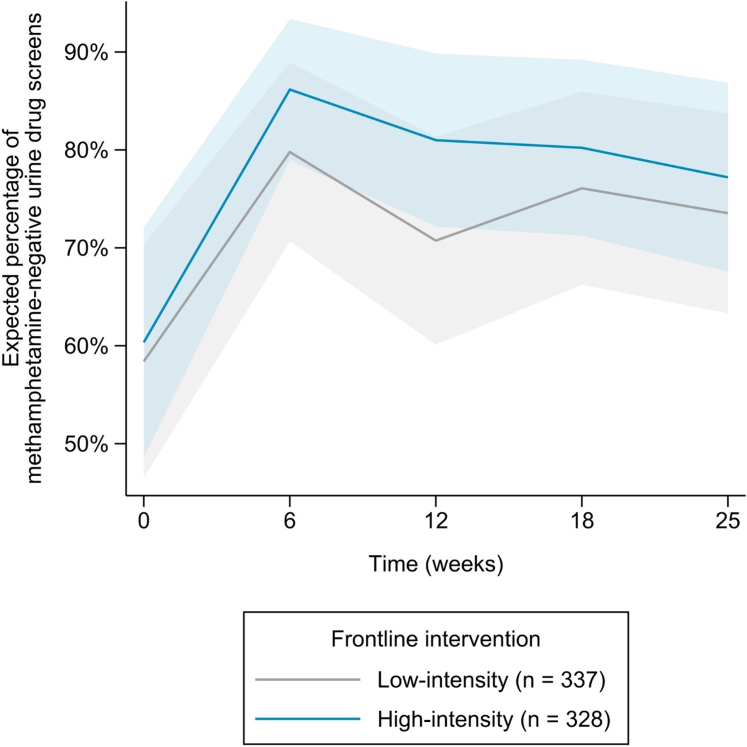
Expected percentage of methamphetamine-negative urine drug screens (with pointwise 95% confidence bands) over the 25-week trial period in low-intensity and high-intensity frontline interventions.

Table 2 displays the expected percentage of MA-negative UDS with 95% confidence intervals (CIs) from baseline (Week 0) to the end of the trial (Week 25) for low-intensity and high-intensity frontline interventions. The time trends for both conditions are plotted in Fig. 2. From baseline to Week 25, the low-intensity condition increased from 58.4% (95% CI [46.5%, 70.3%]) to 73.5% (95% CI [63.3%, 83.7%]), and the high-intensity condition increased from 60.4% (95% CI [48.6%, 72.1%]) to 77.2% (95% CI [67.6%, 86.8%]) (see Table 2). The increase observed in the high-intensity condition by Week 25 was not significantly higher than the low-intensity condition (1.7 percentage points, 95% CI [−1.5%, 4.9%], p = 0.286).

**Table 2:** Expected percentage of methamphetamine-negative urine drug screens and change between time points for frontline interventions and adaptive interventions, marginalized out from mixed effects logistic regression.

	Week	Expected percentage	95% CI^a^		Change^b^	95% CI^a^		p
			Lower	Upper		Lower	Upper	
Frontline intervention								
Low-intensity (n = 337)	0	58.4%	46.5%	70.3%				
	25	73.5%	63.3%	83.7%	15.1%	11.7%	18.5%	<0.001
High-intensity (n = 328)	0	60.4%	48.6%	72.1%				
	25	77.2%	67.6%	86.8%	16.9%	13.1%	20.6%	<0.001
Adaptive Intervention								
Text messaging (n = 428)	14	91.8%	88.6%	94.9%				
	25	88.5%	84.8%	92.2%	−3.3%	−4.9%	−1.6%	<0.001
Matrix only (n = 108)	14	45.1%	33.9%	56.3%				
	25	44.1%	33.1%	55.1%	−1.0%	−4.3%	2.3%	0.553
Matrix + CM (n = 107)	14	54.2%	42.9%	65.4%				
	25	63.2%	52.8%	73.6%	9.1%	5.8%	12.3%	<0.001

a CI = confidence interval.

b Change from Week 0 (baseline) to Week 25 (end of the trial).

The high-intensity condition increased in MA-negative UDS more rapidly than the low-intensity condition (see Fig. 2). By midpoint (Week 12), the high-intensity condition increased by 20.6 percentage points (95% CI [16.2%, 25.1%], p < 0.001), whereas the low-intensity condition increased by 12.3 percentage points (95% CI [9.1%, 15.5%], p < 0.001) (see Fig. 2), a significant difference of 8.3 percentage points (95% CI [4.4%, 12.2%]; p < 0.001).

#### Adaptive intervention effects

Of the 428 participants who had a treatment response during the frontline intervention stage and were reassigned to text messaging during the adaptive intervention stage, MA-negative UDS was 91.8% (95% CI [88.6%, 94.9%]) starting Week 14 and 88.5% (95% CI [84.8%, 92.2%]) ending Week 25, a decline of 3.3 percentage points (95% CI [−4.9%, −1.6%], p < 0.001; Table 2).

**Fig. 3:**
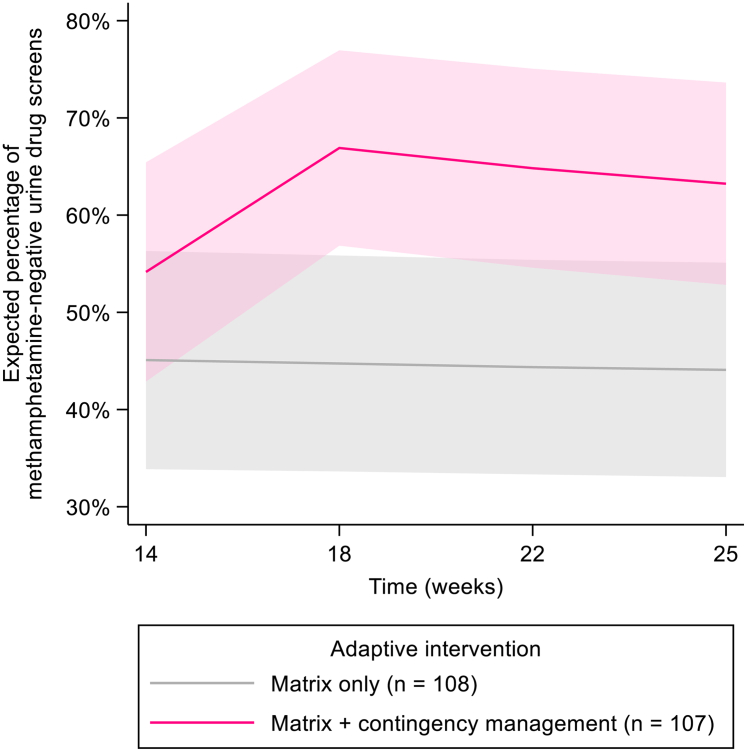
Expected percentage of methamphetamine-negative urine drug screens (with pointwise 95% confidence bands) during the adaptive intervention stage in the Matrix only and Matrix + contingency management conditions.

Table 2 and Fig. 3 display the expected percentage of MA-negative UDS from Weeks 14–25 for participants who did not have a treatment response in the frontline intervention stage and were re-randomized to Matrix only (n = 108) or Matrix + CM (n = 107). MA-negative UDS in the Matrix + CM condition increased from 54.2% (95% CI [42.9%, 65.4%]) at Week 14 to 63.2% (95% CI [52.8%, 73.6%]) at Week 25, a gain of 9.1 percentage points (95% CI [5.8%, 12.3%], p < 0.001). The Matrix only condition did not significantly change.

The joint effects of the frontline and subsequent adaptive interventions are reported in Supplemental Table S1. For each adaptive intervention condition, there were no subgroup differences between previous frontline interventions.

### Secondary outcomes

#### Frontline intervention effects

Of the participants randomized to the high-intensity frontline intervention, 68.6% (95% CI [58.0, 79.2]) achieved a treatment response—four MA-negative UDS out of a possible four—in Weeks 11–12 (see Supplemental Table S2). This was significantly higher—by 11.8 percentage points (95% CI [4.8%, 18.9%], p = 0.001)—than the treatment response in the low-intensity frontline intervention, which was 56.8% (95% CI [45.2%, 68.4%]). By Weeks 24–25, there was no significant difference in treatment response between the high-intensity (57.9%, 95% CI [45.9%, 70.0%]) and low-intensity conditions (54.3%, 95% CI [42.1%, 66.5%]).

In the low-intensity frontline intervention, self-reports of 6–30 days of MA use in the past 30 days decreased by 24.8 percentage points (95% CI [17.7%, 31.9%], p < 0.001) from baseline to Week 13, followed by another 3.1 percentage-points decrease (95% CI [0.4%, 5.9%], p = 0.023) by Week 26 (see Supplemental Figure S1). The high-intensity frontline intervention decreased by 27.5 percentage points (95% CI [19.4%, 35.7%], p < 0.001) in reports of 6–30 days of MA use in the past 30 days by Week 13, then did not significantly change at Week 26.

Self-reported MA use was above the minimum threshold for acceptable sensitivity and specificity when evaluated against urine drug screens.^48^ Of the participants who tested MA-positive on at least one visit during Weeks 9–12, 84.4% (189 out of 224) self-reported using MA one day or more in the past 30 days (i.e., sensitivity). Of participants who tested MA-negative, 90.4% (366 out of 405) self-reported no MA use in the past 30 days (i.e., specificity).

#### Adaptive intervention effects

Of participants re-assigned to text messaging during the adaptive stage, 75.2% (95% CI [66.1%, 84.2%]) had a treatment response in Weeks 24–25 (see Supplemental Table S2). Previous frontline intervention assignment was not significantly associated with having a response during the adaptive intervention stage among those re-assigned to text messaging. Among participants re-assigned to Matrix + CM, 33.3% (95% CI [19.6%, 47.1%]) had a treatment response, significantly greater than the 15.9% (95% CI [6.0%, 25.8%]) in the Matrix only condition by 17.4 percentage points (95% CI [5.1%, 29.7%], p = 0.005; Supplemental Table S2).

From Week 13–26, both Matrix only and Matrix + CM conditions decreased in self-reports of 6–30 days of MA use in the past 30 days (see Supplemental Figure S2). The Matrix only condition decreased by 11.7 percentage-points (95% CI [3.4%, 20.0%], p = 0.006), while the Matrix + CM condition decreased by 14.8 percentage-points (95% CI [7.0%, 22.6%], p < 0.001).

#### Non-response and adverse events

Overall, 196 participants (29.5%) persistently did not meet treatment response criteria. This means they tested MA-positive at least once during both Weeks 11–12 (end of the frontline intervention) *and* Weeks 24–25 (end of the adaptive intervention).

Information about adverse events were collected directly from participants and from providers and family members (see Supplemental Table S3). Moderate severity cases included eight COVID-19 infections, four accidents, and four mental health problems, all of which eventually resolved. Nonfatal serious adverse events included 13 incarcerations, 11 compulsory drug rehabilitation placements, and seven hospitalizations. There were three deaths during the frontline stage caused by kidney failure, asthma, and non-HIV-related pneumonia, and three deaths during the adaptive stage caused by bowel obstruction, suicide possibly MA-related, and pleural effusion. No adverse events were attributed to participation.

## Discussion

The STAR-OM trial provides evidence for the use of adaptive behavioral interventions in treatment models that address the opioid-stimulant co-epidemic in Vietnam.^3^^,^^10^^,^^11^ Both frontline interventions reduced MA use, but the high-intensity condition showed a steeper change than the low-intensity condition through Week 6 and maintained lower use than the low-intensity condition through Week 12. Participants who met treatment response criteria at Week 12 maintained reductions in MA use while receiving text messaging, with MA-negative UDS remaining between 92% at Week 14 and 89% at Week 25; although statistically significant, the loss of treatment gains was marginal.

Among MMT patients who did not respond to the first 12 weeks of intervention, providing Matrix combined with CM for another 12 weeks led to further decreases in MA use, while Matrix only yielded no further change. This aligns with U.S. research indicating that the effects of CM can be augmented when combined with another psychosocial intervention (e.g., cognitive behavioral therapy).^15^ Furthermore, among patients who respond early to treatment, unidirectional, theory-based text messaging appears to support behavioral maintenance.

Findings suggest that evidence-based behavioral interventions implemented in MMT clinics may be effective in reducing stimulant co-use. Findings also underscore the value of tailoring intervention sequences to patient responses in real-world clinical settings, offering an example of an integrated approach for addressing polysubstance use in low- and middle-income countries, as well as in countries with MMT clinics impacted by polysubstance use, such as the United States^13^ and Canada.^14^

About 30% of participants did not meet the strict criteria for a treatment response in either intervention stage. This threshold was intentionally conservative, ensuring adequate sample sizes in the adaptive intervention stage and identifying those who, despite showing progress, had not yet fully stabilized their MA use and could benefit from augmented intervention. Self-reports of 6–30 days of MA use in the past 30 days decreased, suggesting partial or incremental effects that remain clinically meaningful, as lower frequency of MA use is associated with reduced risk of psychosocial issues, infectious diseases, and psychiatric and chronic health conditions.^4^

The strengths of this trial include high retention (90%), representation across 15 MMT clinics in Vietnam, twice-weekly UDS, and a SMART design to model real-world adaptive treatment scenarios. To our knowledge, this is among the first trials to use an adaptive behavioral intervention design to reduce MA use in MMT settings.

This trial has limitations. Although retention was high (90%), dropout may not have been missing at random, as continued MA use may have led to life events preventing trial participation, potentially overestimating intervention effects in affected conditions. All mixed models were estimated via maximum likelihood, incorporating all available observations from all randomized participants under the intent-to-treat principle to reduce the impact of missing data on inference.^42^ Findings may not generalize beyond Hanoi and Ho Chi Minh City, to other healthcare systems or models of opioid agonist treatment (e.g., buprenorphine), to populations not receiving MMT, or to populations formally diagnosed with MA use disorder. Few women enrolled (11%) due to low substance use and MMT participation among women in Vietnam.^3^^,^^10^^,^^11^

The STAR-OM trial suggests that consistent reinforcement with CM may support greater reductions in MA use than other behavioral interventions in patients on MMT for OUD, that CM can be augmented with another intervention in those who do not initially respond to treatment, and that theory-based text messaging can support behavioral maintenance in people with early reductions in MA use. This trial provides an example for implementing evidence-based behavioral interventions at MMT clinics in Vietnam to address polysubstance use. Future research is needed to evaluate implementation in other settings, including MMT clinics in rural settings, other opioid agonist treatment models, and primary care settings as well as among populations with primary MA use disorder.

## Contributors

All authors have reviewed and approved the final version of the manuscript. Below is a summary of specific contributions to this study by each author:

• Le Minh Giang—conceptualization, funding acquisition, methodology, project administration, resources, supervision, validation, writing (original draft preparation), writing (review and edit).
• Michael J. Li—data curation, formal analysis, methodology, visualization, writing (original draft preparation), writing (review and edit).
• Nguyen Thu Trang—data curation, investigation, project administration, supervision, visualization, validation, writing (original draft preparation), writing (review and edit).
• Nguyen Bich Diep—data curation, investigation, project administration, supervision, visualization, validation, writing (original draft preparation), writing (review and edit).
• Dao Thi Dieu Thuy—investigation, writing (review and edit).
• Dinh Thanh Thuy—investigation, writing (review and edit).
• Han Dinh Hoe– investigation, writing (review and edit).
• Hoang Thi Hai Van—data curation, investigation, methodology, validation, writing (review and edit).
• Thai Thanh Truc—data curation, investigation, methodology, validation, writing (review and edit).
• Hoa Hong Nguyen—investigation, methodology, writing (review and edit).
• Nguyen Ly Lai—investigation, resources, writing (review and edit).
• Pham Thi Dan Linh—investigation, resources, writing (review and edit).
• Vu Thi Tuong Vi—investigation, resources, writing (review and edit).
• Cathy J. Reback—conceptualization, methodology, writing (review and edit).
• Arleen Leibowitz—conceptualization, methodology, writing (review and edit).
• Li Li—conceptualization, methodology, validation, writing (review and edit)
• Chunqing Lin—conceptualization, methodology, validation, writing (review and edit).
• Do Van Dung—conceptualization, methodology, supervision, writing (review and edit).
• Steven J. Shoptaw—conceptualization, funding acquisition, methodology, project administration, supervision, validation, writing (review and edit).

## Data sharing statement

Any de-identified participant-level data collected during the trial, along with a data dictionary, will be made available upon approval of a submitted concept proposal beginning 12 months after publication of this article and ending 5 years after publication of this article. Requests should include a concept sheet describing the proposed use of the data and may be submitted to leminhgiang@hmu.edu.vn. Proposals will be reviewed by the investigative team, and data sharing will be limited to variables relevant to the approved proposal. Approved requestors will be required to sign a data access agreement, after which data will be shared via a secure, HIPAA-compliant file transfer platform.

## Declaration of interests

Dr. Steven Shoptaw's institution, the University of California, Los Angeles, has received clinical supplies from Alkermes, Gilead Sciences, and Indivior for his research. Dr. Shoptaw has received consulting honoraria from Clear Scientific, Inc. and Lilly, Inc. on medication development and payment by Lilly, Inc. for participation on a Data Safety Monitoring Board. All other authors declare no conflicts of interest.

## Appendix A Supplementary data

Supplemental Table S3

Trials - Published Protocol

## References

[1] Han B., Compton W.M., Jones C.M., Einstein E.B., Volkow N.D. (2021). Methamphetamine use, methamphetamine use disorder, and associated overdose deaths among US adults. JAMA Psychiatry.

[2] Ellis M.S., Kasper Z.A., Cicero T.J. (2018). Twin epidemics: the surging rise of methamphetamine use in chronic opioid users. Drug Alcohol Depend.

[3] Des Jarlais D.C., Feelemyer J., Arasteh K. (2021). The methamphetamine epidemic among persons who inject heroin in Hai Phong, Vietnam. J Subst Abuse Treat.

[4] Shoptaw S., Li M.J., Javanbakht M., Ragsdale A., Goodman-Meza D., Gorbach P.M. (2022). Frequency of reported methamphetamine use linked to prevalence of clinical conditions, sexual risk behaviors, and social adversity in diverse men who have sex with men in Los Angeles. Drug Alcohol Depend.

[5] Shearer R.D., Howell B.A., Bart G., Winkelman T.N.A. (2020). Substance use patterns and health profiles among US adults who use opioids, methamphetamine, or both, 2015-2018. Drug Alcohol Depend.

[6] Feelemyer J., Arasteh K., Huong D.T. (2020). Associations between methamphetamine use and lack of viral suppression among a cohort of HIV-positive persons who inject drugs in Hai Phong, Vietnam. AIDS.

[7] Li M.J., Su E., Garland W.H. (2020). Trajectories of viral suppression in people living with HIV receiving coordinated care: differences by comorbidities. J Acquir Immune Defic Syndr.

[8] Diep N.B., Trang N.T., Huy D.D. (2025). Non-adherence to treatment and concurrent opioid use among people on methadone maintenance treatment using methamphetamine in Vietnam. J Subst Use Addict Treat.

[9] Jones C.M., Underwood N., Compton W. (2019). Increases in methamphetamine use among heroin treatment admissions in the United States, 2008-2017. Addiction.

[10] Le N.T., Khuong Q.L., Vu T.T.V. (2021). Prevalence of amphetamine-type stimulant use and related factors among methadone maintenance patients in Ho Chi Minh City Vietnam: a cross-sectional study. J Psychoactive Drugs.

[11] Giang L.M., Li M.J., Okafor C.N., Diep N.B., Shoptaw S.J. (2022). Correlates of methamphetamine use severity among patients receiving methadone maintenance treatment for opioid use disorder in Vietnam. J Subst Abuse Treat.

[12] Hoang T., Nguyen H., Shiraishi R.W. (2018). Factors associated with concurrent heroin use among patients on methadone maintenance treatment in Vietnam: a 24-month retrospective analysis of a nationally representative sample. Int J Drug Policy.

[13] Jones C.M., Houry D., Han B., Baldwin G., Vivolo-Kantor A., Compton W.M. (2022). Methamphetamine use in the United States: epidemiological update and implications for prevention, treatment, and harm reduction. Ann N Y Acad Sci.

[14] Cui Z., Bach P., Ti L. (2022). Opioid agonist therapy engagement and crystal methamphetamine use: the impact of unregulated opioid use in Vancouver, Canada. Int J Drug Policy.

[15] Shoptaw S., Reback C.J., Peck J.A. (2005). Behavioral treatment approaches for methamphetamine dependence and HIV-related sexual risk behaviors among urban gay and bisexual men. Drug Alcohol Depend.

[16] Knight R., Karamouzian M., Carson A. (2019). Interventions to address substance use and sexual risk among gay, bisexual and other men who have sex with men who use methamphetamine: a systematic review. Drug Alcohol Depend.

[17] AshaRani P.V., Hombali A., Seow E., Ong W.J., Tan J.H., Subramaniam M. (2020). Non-pharmacological interventions for methamphetamine use disorder: a systematic review. Drug Alcohol Depend.

[18] Polcin D.L., Bond J., Korcha R., Nayak M.B., Galloway G.P., Evans K. (2014). Randomized trial of intensive motivational interviewing for methamphetamine dependence. J Addict Dis.

[19] Brown H.D., DeFulio A. (2020). Contingency management for the treatment of methamphetamine use disorder: a systematic review. Drug Alcohol Depend.

[20] Shoptaw S., Klausner J.D., Reback C.J. (2006). A public health response to the methamphetamine epidemic: the implementation of contingency management to treat methamphetamine dependence. BMC Public Health.

[21] Verstraete A.G. (2004). Detection times of drugs of abuse in blood, urine, and oral fluid. Ther Drug Monit.

[22] Petry N.M., Alessi S.M., Olmstead T.A., Rash C.J., Zajac K. (2017). Contingency management treatment for substance use disorders: how far has it come, and where does it need to go?. Psychol Addict Behav.

[23] Ginley M.K., Pfund R.A., Rash C.J., Zajac K. (2021). Long-term efficacy of contingency management treatment based on objective indicators of abstinence from illicit substance use up to 1 year following treatment: a meta-analysis. J Consult Clin Psychol.

[24] Radfar S.R., Rawson R.A. (2014). Current research on methamphetamine: epidemiology, medical and psychiatric effects, treatment, and harm reduction efforts. Addict Health.

[25] Rawson R.A., Marinelli-Casey P., Anglin M.D. (2004). A multi-site comparison of psychosocial approaches for the treatment of methamphetamine dependence. Addiction.

[26] Reback C.J., Fletcher J.B., Swendeman D.A., Metzner M. (2019). Theory-based text-messaging to reduce methamphetamine use and HIV sexual risk behaviors among men who have sex with men: automated unidirectional delivery outperforms bidirectional peer interactive delivery. AIDS Behav.

[27] Reback C.J., Fletcher J.B., Shoptaw S., Mansergh G. (2015). Exposure to theory-driven text messages is associated with HIV risk reduction among methamphetamine-using men who have sex with men. AIDS Behav.

[28] Reback C.J., Fletcher J.B., Kisler K.A. (2021). Text messaging improves HIV care continuum outcomes among young adult trans women living with HIV: text Me, Girl. AIDS Behav.

[29] Reback C.J., Fletcher J.B., Leibowitz A.A. (2019). Cost effectiveness of text messages to reduce methamphetamine use and HIV sexual risk behaviors among men who have sex with men. J Subst Abuse Treat.

[30] Paulus M.P., Stewart J.L. (2020). Neurobiology, clinical presentation, and treatment of methamphetamine use disorder: a review. JAMA Psychiatry.

[31] Li M.J., Shoptaw S.J. (2023). Clinical management of psychostimulant withdrawal: review of the evidence. Addiction.

[32] Murphy S.A., Lynch K.G., Oslin D., McKay J.R., TenHave T. (2007). Developing adaptive treatment strategies in substance abuse research. Drug Alcohol Depend.

[33] Giang L.M., Trang N.T., Diep N.B. (2022). An adaptive design to screen, treat, and retain people with opioid use disorders who use methamphetamine in methadone clinics (STAR-OM): study protocol of a clinical trial. Trials.

[34] Giang L.M., Trang N.T., Thuy D.T. (2023). Using ADAPT-ITT framework to tailor evidence-based interventions for addressing methamphetamine use among methadone patients in Vietnam. Drug Alcohol Rev.

[35] Humeniuk R., Ali R., Babor T.F. (2008). Validation of the alcohol, smoking and substance involvement screening test (ASSIST). Addiction.

[36] Infographics - Vietnam News Agency (2025).

[37] Nguyen T.H., Nguyen T.T., Dao T.D.T. (2025). Operationalizing contingency management to improve adherence and retention in methadone treatment: a scoping review. Subst Use Misuse.

[38] Vaux A. (1988).

[39] Bandura A. (2001). Social cognitive theory: an agentic perspective. Annu Rev Psychol.

[40] Janz N.K., Becker M.H. (1984). The health belief model: a decade later. Health Educ Q.

[41] Rabe-Hesketh S., Skrondal A. (2022).

[42] Enders C.K. (2001). A primer on maximum likelihood algorithms available for use with missing data. Struct Equ Model.

[43] Burnham K.P., Anderson D.R. (2004). Multimodel inference: understanding AIC and BIC in model selection. Sociol Methods Res.

[44] Williams R. (2012). Using the margins command to estimate and interpret adjusted predictions and marginal effects. STATA J.

[45] Wagner A.K., Soumerai S.B., Zhang F., Ross-Degnan D. (2002). Segmented regression analysis of interrupted time series studies in medication use research. J Clin Pharm Ther.

[46] Cleveland W.S. (1979). Robust locally weighted regression and smoothing scatterplots. J Am Stat Assoc.

[47] Collins A.B., Strike C., Guta A. (2017). “We’re giving you something so we get something in return”: perspectives on research participation and compensation among people living with HIV who use drugs. Int J Drug Policy.

[48] Power M., Fell G., Wright M. (2013). Principles for high-quality, high-value testing. Evid Based Med.

