# Supplementary material for: Interventions for methamphetamine use among people on methadone maintenance treatment in Vietnam: a sequential multiple assignment randomized trial (STAR-OM)
Source: Lancet Reg Health Southeast Asia. 2026 Apr 24;48:100773. doi: 10.1016/j.lansea.2026.100773 (PMC13129376; doi:10.1016/j.lansea.2026.100773)
Supplement: Supplemental Figure S1 [file mmc1.docx]

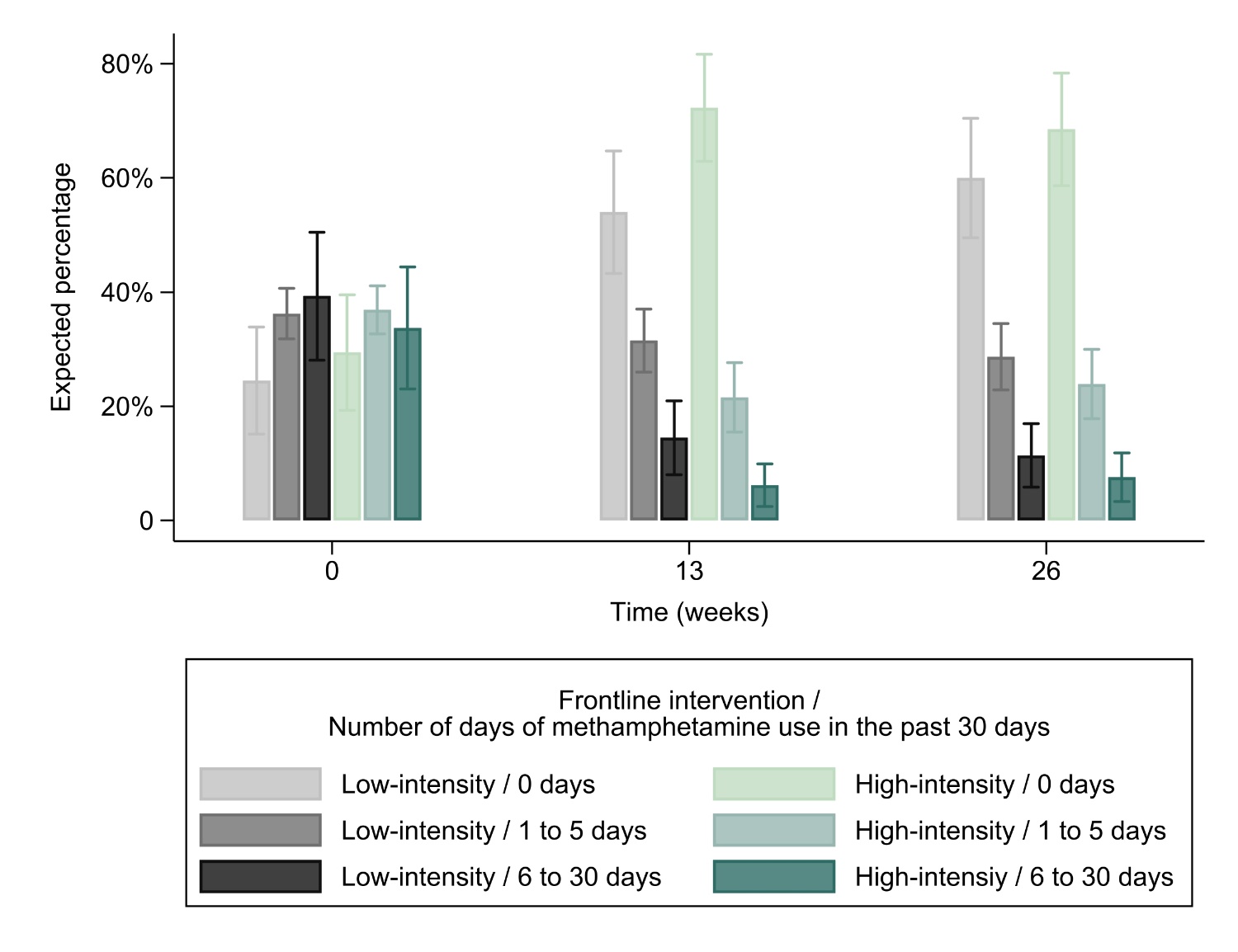


Supplemental Figure S1. Expected percentage (with 95% confidence intervals) of self-reporting 0, 1 to 5 days, and 6 to 30 days of methamphetamine use over time in low-intensity (n = 337) and high-intensity frontline intervention conditions (n = 328).
