# Supplementary material for: Interventions for methamphetamine use among people on methadone maintenance treatment in Vietnam: a sequential multiple assignment randomized trial (STAR-OM)
Source: Lancet Reg Health Southeast Asia. 2026 Apr 24;48:100773. doi: 10.1016/j.lansea.2026.100773 (PMC13129376; doi:10.1016/j.lansea.2026.100773)
Supplement: Supplemental Table S1 [file mmc3.docx]

**Supplemental Table S1. Expected percentage of methamphetamine-negative urine drug screens and change between time points during the adaptive intervention stage, stratified by frontline intervention assignment and subsequent adaptive intervention assignment**

|  |  |  | 95% CI ^a^ | |  | 95% CI ^a^ | |  |
| --- | --- | --- | --- | --- | --- | --- | --- | --- |
| Intervention | Week | Expected percentage | Lower | Upper | Change ^b^ | Lower | Upper | p |
| Low-intensity / text messaging (n = 198) | 14 | 92.4% | 89.0% | 95.8% |  |  |  |  |
|  | 25 | 89.3% | 85.2% | 93.3% | -3.1% | -5.5% | -0.8% | 0.008 |
| High-intensity / text messaging (n = 230) | 14 | 91.3% | 87.8% | 94.8% |  |  |  |  |
|  | 25 | 87.9% | 83.8% | 92.1% | -3.4% | -5.6% | -1.2% | 0.003 |
| Low-intensity / Matrix only (n = 68) | 14 | 40.1% | 28.3% | 51.9% |  |  |  |  |
|  | 25 | 40.3% | 28.7% | 51.8% | 0.2% | -3.8% | 4.2% | 0.926 |
| Low-intensity / Matrix + CM (n = 62) | 14 | 55.8% | 43.8% | 67.8% |  |  |  |  |
|  | 25 | 65.2% | 54.3% | 76.1% | 9.4% | 5.2% | 13.6% | < 0.001 |
| High-intensity / Matrix only (n = 40) | 14 | 54.6% | 41.2% | 68.0% |  |  |  |  |
|  | 25 | 51.3% | 38.2% | 64.4% | -3.3% | -9.3% | 2.7% | 0.286 |
| High-intensity / Matrix + CM (n = 42) | 14 | 51.8% | 38.6% | 65.1% |  |  |  |  |
|  | 25 | 60.6% | 48.3% | 72.9% | 8.8% | 4.0% | 13.5% | < 0.001 |

Notes. ^a^ CI = confidence interval; ^b^ Change from Week 14 (start of the adaptive stage) to Week 25 (end of the trial)
