# Supplementary material for: Interventions for methamphetamine use among people on methadone maintenance treatment in Vietnam: a sequential multiple assignment randomized trial (STAR-OM)
Source: Lancet Reg Health Southeast Asia. 2026 Apr 24;48:100773. doi: 10.1016/j.lansea.2026.100773 (PMC13129376; doi:10.1016/j.lansea.2026.100773)
Supplement: Supplemental Table S2 [file mmc4.docx]

**Supplemental Table S2. Expected percentage of achieving a treatment response in the frontline intervention stage and the adaptive intervention stage between interventions**

|  |  | 95% CI ^a^ | |  | 95% CI ^a^ | |  |
| --- | --- | --- | --- | --- | --- | --- | --- |
|  | Expected percentage | Lower | Upper | Difference | Lower | Upper | p |
| Frontline intervention stage (Weeks 11-12) | | | | | | | |
| Low-intensity | 56.8% | 45.2% | 68.4% |  |  |  |  |
| High-intensity | 68.6% | 58.0% | 79.2% | 11.8% ^b^ | 4.8% | 18.9% | 0.001 |
| End of trial (Weeks 24-25), by frontline intervention | | | | | | | |
| Low-intensity | 54.3% | 42.1% | 66.5% |  |  |  |  |
| High-intensity | 57.9% | 45.9% | 70.0% | 1.7% ^b^ | -1.5% | 4.9% | 0.313 |
| Adaptive intervention stage (Weeks 24-25) | | | | | | | |
| Text messaging | 75.2% | 66.1% | 84.2% |  |  |  |  |
| Matrix | 15.9% | 6.0% | 25.8% |  |  |  |  |
| Matrix + CM | 33.3% | 19.6% | 47.1% | 17.4% ^c^ | 5.1% | 29.7% | 0.005 |

Notes. ^a^ CI = confidence interval; ^b^ Difference in treatment response compared to the low-intensity frontline intervention;

^c^ Difference in treatment response compared to the Matrix only adaptive intervention
