# Supplementary material for: Interventions for methamphetamine use among people on methadone maintenance treatment in Vietnam: a sequential multiple assignment randomized trial (STAR-OM)
Source: Lancet Reg Health Southeast Asia. 2026 Apr 24;48:100773. doi: 10.1016/j.lansea.2026.100773 (PMC13129376; doi:10.1016/j.lansea.2026.100773)
Supplement: Supplemental Table S3 [file mmc5.docx]

**Supplemental Table S3. Frequency of adverse events in each intervention condition**

|  | Frontline intervention stage | | Adaptive intervention stage | | | | | |
| --- | --- | --- | --- | --- | --- | --- | --- | --- |
| Events | Low intensity  (n = 337) | High intensity  (n = 328) | Low intensity / text messaging  (n = 198) | Low intensity / Matrix only  (n = 68) | Low intensity / Matrix + contingency management  (n = 62) | High intensity / text messaging  (n = 230) | High intensity / Matrix only  (n = 40) | High intensity / Matrix + contingency management  (n = 45) |
|  | n (%) | n (%) | n (%) | n (%) | n (%) | n (%) | n (%) | n (%) |
| Adverse events |  |  |  |  |  |  |  |  |
| COVID-19 infection (mild to moderate) | 4 | 2 |  | 2 |  |  |  |  |
| Accidental injury |  |  |  |  |  |  |  |  |
| Automotive accident |  |  | 1 | 1 |  | 1 |  |  |
| Sprained foot |  |  |  |  |  |  | 1 |  |
| Mental health problems |  | 2 |  |  |  |  | 1 | 1 |
| Serious adverse events |  |  |  |  |  |  |  |  |
| Incarceration | 4 | 3 | 2 |  |  |  | 2 | 2 |
| Sent to compulsory drug rehabilitation programs | 3 | 2 |  | 1 |  | 2 | 2 | 1 |
| Hospitalization |  |  |  |  |  |  |  |  |
| Psychosis |  |  |  |  |  | 1 |  | 1 |
| Bowel obstruction | 1 |  |  |  |  |  |  |  |
| Automotive accident |  |  |  |  |  | 1 |  |  |
| Bowel infection | 2 |  |  |  |  |  | 1 |  |
|  |  |  |  |  |  |  |  |  |
| Death |  |  |  |  |  |  |  |  |
| Kidney failure |  | 1 |  |  |  |  |  |  |
| Asthma |  | 1 |  |  |  |  |  |  |
| Non-HIV-related pneumonia | 1 |  |  |  |  |  |  |  |
| Bowel obstruction |  |  | 1 |  |  |  |  |  |
| Suicide |  |  |  |  |  |  | 1 |  |
| Pleural effusion |  |  | 1 |  |  |  |  |  |
