# Supplementary material for: Interventions for methamphetamine use among people on methadone maintenance treatment in Vietnam: a sequential multiple assignment randomized trial (STAR-OM)
Source: Lancet Reg Health Southeast Asia. 2026 Apr 24;48:100773. doi: 10.1016/j.lansea.2026.100773 (PMC13129376; doi:10.1016/j.lansea.2026.100773)
Supplement: Trials - Published Protocol [file mmc6.pdf]

Giang et al. Trials (2022) 23:342
https://doi.org/10.1186/s13063-022-06278-w

Trials

STUDY PROTOCOL Open Access

An adaptive design to screen, treat, and retain people with opioid use disorders who use methamphetamine in methadone clinics (STAR-OM): study protocol of a clinical trial

Le Minh Giang[1], Nguyen Thu Trang[1*], Nguyen Bich Diep[1], Dao Thi Dieu Thuy[1], Dinh Thanh Thuy[1], Han Dinh Hoe[1], Hoang Thi Hai Van[2], Thai Thanh Truc[3], Hoa H. Nguyen[4], Nguyen Ly Lai[5], Pham Thi Dan Linh[5], Vu Thi Tuong Vi[5], Cathy J. Reback[6], Arleen Leibowitz[7], Li Li[8], Chunqing Lin[9], Michael Li[10], Do Van Dung[3] and Steve Shoptaw[10]

Abstract

Background: Methamphetamine use could jeopardize the current efforts to address opioid use disorder and HIV infection. Evidence-based behavioral interventions (EBI) are effective in reducing methamphetamine use. However, evidence on optimal combinations of EBI is limited. This protocol presents a type-1 effectiveness-implementation hybrid design to evaluate the effectiveness, cost-effectiveness of adaptive methamphetamine use interventions, and their implementation barriers in Vietnam.

Method: *Design:* Participants will be first randomized into two frontline interventions for 12 weeks. They will then be placed or randomized to three adaptive strategies for another 12 weeks. An economic evaluation and an ethnographic evaluation will be conducted alongside the interventions.

Participants: We will recruit 600 participants in 20 methadone clinics. Eligibility criteria: (1) age 16+; (2) Alcohol, Smoking and Substance Involvement Screening Test (ASSIST) scores ≥ 10 for methamphetamine use or confirmed methamphetamine use with urine drug screening; (3) willing to provide three pieces of contact information; and (4) having a cell phone.

Outcomes: Outcomes are measured at 13, 26, and 49 weeks and throughout the interventions. Primary outcomes include the (1) increase in HIV viral suppression, (2) reduction in HIV risk behaviors, and (3) reduction in methamphetamine use.

COVID-19 response: We developed a response plan for interruptions caused by COVID-19 lockdowns to ensure data quality and intervention fidelity.

* Correspondence: trangthu@hmu.edu.vn
[1]Centre for Training and Research on Substance use and HIV, Hanoi Medical University, Hanoi, Vietnam
Full list of author information is available at the end of the article

© The Author(s). 2022 **Open Access** This article is licensed under a Creative Commons Attribution 4.0 International License, which permits use, sharing, adaptation, distribution and reproduction in any medium or format, as long as you give appropriate credit to the original author(s) and the source, provide a link to the Creative Commons licence, and indicate if changes were made. The images or other third party material in this article are included in the article's Creative Commons licence, unless indicated otherwise in a credit line to the material. If material is not included in the article's Creative Commons licence and your intended use is not permitted by statutory regulation or exceeds the permitted use, you will need to obtain permission directly from the copyright holder. To view a copy of this licence, visit http://creativecommons.org/licenses/by/4.0/. The Creative Commons Public Domain Dedication waiver (http://creativecommons.org/publicdomain/zero/1.0/) applies to the data made available in this article, unless otherwise stated in a credit line to the data.

Discussion: This study will provide important evidence for scale-up of EBIs for methamphetamine use among methadone patients in limited-resource settings. As the EBIs will be delivered by methadone providers, they can be readily implemented if the trial demonstrates effectiveness and cost-effectiveness.

Trial registration: ClinicalTrials.gov NCT04706624. Registered on 13 January 2021. https://clinicaltrials.gov/ct2/show/NCT04706624

Keywords: Methamphetamine, Amphetamine-type stimulants, Methadone, Opioid substitution, Vietnam, Randomized controlled trial

Introduction

The global rise of methamphetamine use could jeopardize current intervention efforts to address the twin epidemics of opioid use disorder (OUD) and HIV infection. Use of methamphetamine is increasingly common among people with primary OUD [1–3]. Prevalence of methamphetamine use disorders is increasing in Vietnam [4], raising concerns about increased risk of HIV infection [5–8] and disruption of the substance use treatment systems, especially methadone programs [9, 10]. Methamphetamine use among people living with HIV could decrease retention in care, hinder medication adherence, accelerate viral replication, and further HIV disease progression [11–15]. Other countries beyond South-East Asia encounter similar challenges [16–18]. In low-and-middle-income countries, it is vital to identify cost-effective models of adapted evidence-based practices for addressing substance use disorders [19, 20].

Although there are no approved pharmacological treatments for methamphetamine use, evidence-based behavioral interventions (EBI) such as motivational interviewing, contingency management, and cognitive behavioral therapy, including Matrix model, have shown efficacy in reducing methamphetamine use [21–23]. However, we need to identify optimal combinations of EBI for effectiveness and cost-effectiveness as many people in treatment face challenges to retention and sustained reductions in use.

Motivational interviewing

Motivational interviewing helps individuals to evaluate the pros and cons to change drug use and to develop personalized change behaviors. Motivational interviewing can be used in a single session or in multiple sessions [22]. Polcin et al. [24] compared two motivational interviewing conditions (9 sessions vs. 1 session) and found that both groups showed significant reductions in methamphetamine use without differences between the two groups [24]. A greater reduction in psychiatric symptoms including anxiety and depression was found among those receiving more motivational interviewing sessions [21, 24, 25].

Contingency management

Contingency management has shown the strongest evidence in treating methamphetamine use disorders [21, 26–30]. It is also effective in reducing other drug use including alcohol, cannabis, nicotine, and opioids [31]. Contingency management is based on the theory of operant conditioning where incentives are used to strengthen the target behavior such as abstinence, reduction of sexual risk behaviors, or other health-promoting behaviors like retention or adherence to treatment [27]. Contingency management effects are enhanced in combination with other psychosocial interventions or education [22]. A recent meta-analysis shows contingency management is more efficacious than other EBI up to 1 year following the discontinuation of reinforcers [32].

Matrix model

The Matrix model has shown greater reduction in methamphetamine use, risky behaviors, and more days of abstinence compared to non-standardized outpatient treatment approaches [21, 23]. This intervention combines different elements of effective approaches including cognitive and behavioral treatment using accurate information on the effects of stimulants, relapse prevention skills training, 12-step program participation, and family education [33]. Its manualized treatment protocol ensures fidelity when the model is implemented in different settings.

SMS text messages

Using SMS text messages with people who use methamphetamine has been shown to reduce methamphetamine use and HIV-related sexual transmission behaviors [34, 35] and increase retention in HIV care among some key populations [36]. Scripted unidirectional texts outperform bidirectional interactive text-messaging conversations in reducing methamphetamine use and HIV sexual risk behaviors and are more cost-effective than in-person therapies [37]. Theory-driven messaging might better benefit people in the early stages of behavior change (e.g., non-treatment seeking participants) than people who are already seeking help [34].

Despite some demonstrated efficacy, few studies have shown ways to optimize and combine treatment approaches for methamphetamine use disorders. Qualitative reports show patients found contingency management beneficial when combined with motivational interviewing and cognitive behavioral techniques for methamphetamine use disorders [38]. Combined motivational interviewing and cognitive behavioral treatment show efficacy in reducing methamphetamine use in HIV-positive MSM [39]. Evidence supports combining psychosocial treatment with medication-assisted treatment in people with OUD [40], but it is unclear whether patients with comorbid methamphetamine use disorder will experience similar benefits.

Integrating screening and brief interventions [41–43], contingency management or conditional cash transfer [6, 44, 45], and cognitive behavioral therapy [46–48] for the management of substance use disorders requires trained health professionals. This is challenging in settings where human resource for mental health/substance use is scarce. Therefore, besides identifying optimal combination of EBI, it is essential to recognize potential barriers to the implementation of these strategies. Our study named "Screen, Treat and Retain people with opioid use disorders who use methamphetamine in methadone clinics" (STAR-OM) proposes to explore these questions.

The study has three aims:

Aim 1: To develop and to compare the effectiveness of two frontline interventions and four adaptive strategies in improving HIV and substance use outcomes among people with OUD who use methamphetamine at methadone clinics in the two largest cities in Vietnam.

Aim 2: To compare cost-effectiveness of two frontline interventions and of four adaptive strategies in improving both HIV and substance use outcomes among people with OUD who use methamphetamine at methadone clinics.

Aim 3: To identify the structural, provider, and patient-level factors that influence adoption and scale-up of the studied model in methadone clinics.

Method

Overview of the trial design

The study deploys a type-1 effectiveness-implementation hybrid design to evaluate the effectiveness of the proposed adaptive interventions and gather data on the implementation [49]. To evaluate the effectiveness of the interventions, the study employs a Sequential Multiple Assignment Randomized Trial (SMART) design. In the first phase, participants will be randomized into two frontline interventions for 12 weeks. Based on their outcome at the end of this phase, they will be placed or randomized into three adaptive strategies for another 12 weeks (Fig. 1). The economic evaluation that addresses Aim 2 aims to weigh public health and societal costs against public health and societal benefits attributed to the interventions of different intensities with a time horizon of 12 months. To address Aim 3, we will conduct an ethnographic evaluation to identify the multi-level factors that influence the adoption and scale-up of the interventions in methadone clinics. The ethnographic evaluation is guided by the Consolidated Framework for Implementation Research (CFIR) [50, 51]. The CFIR assesses five domains of interventions, outer settings, inner settings, provider characteristics, and participant characteristics. The evaluation includes pre- and post-intervention in-depth interviews with key informants who participate in the study and ethnographic observation with participants in their daily activities at the clinics and in the community settings.

Settings

Participants are recruited from the methadone clinics in Hanoi and Ho Chi Minh City (HCMC)—the two largest urban settings in Vietnam. As of March 2021, there are 23 methadone clinics in HCMC and 18 in Hanoi, treating 5047 and 4655 patients, respectively [52, 53]. Criteria for selecting clinics include number of patients, estimated prevalence of methamphetamine use, availability of human resources to implement study interventions, and space for intervention activities. Ten clinics in each city will be randomly selected from those that meet the criteria.

Study schedule

The study pilot phase started in November 2020. The full study implementation phase began in May 2021. To recruit 200 HIV-positive and 400 HIV-negative methamphetamine-using methadone participants from both cities, we will screen about 5000 patients in 20 methadone clinics. A cluster of four methadone clinics will start every 6 months. We expect to complete the last cluster in October 2023.

Participants

Participants under methadone treatment

We will recruit 600 methadone participants with the following eligibility criteria: (1) age 16 or older; (2) Alcohol, Smoking and Substance Involvement Screening Test (ASSIST) scores 10 or more for methamphetamine use or confirmed methamphetamine use with urine drug screening (UDS); (3) willing to provide at least three pieces of contact information; and (4) has a cell phone that can receive text messages. The criteria of ASSIST scores and UDS had been modified after the pilot implementation (see Modification of eligibility criteria). Exclusion criteria are as follows: (1) psychosis or other

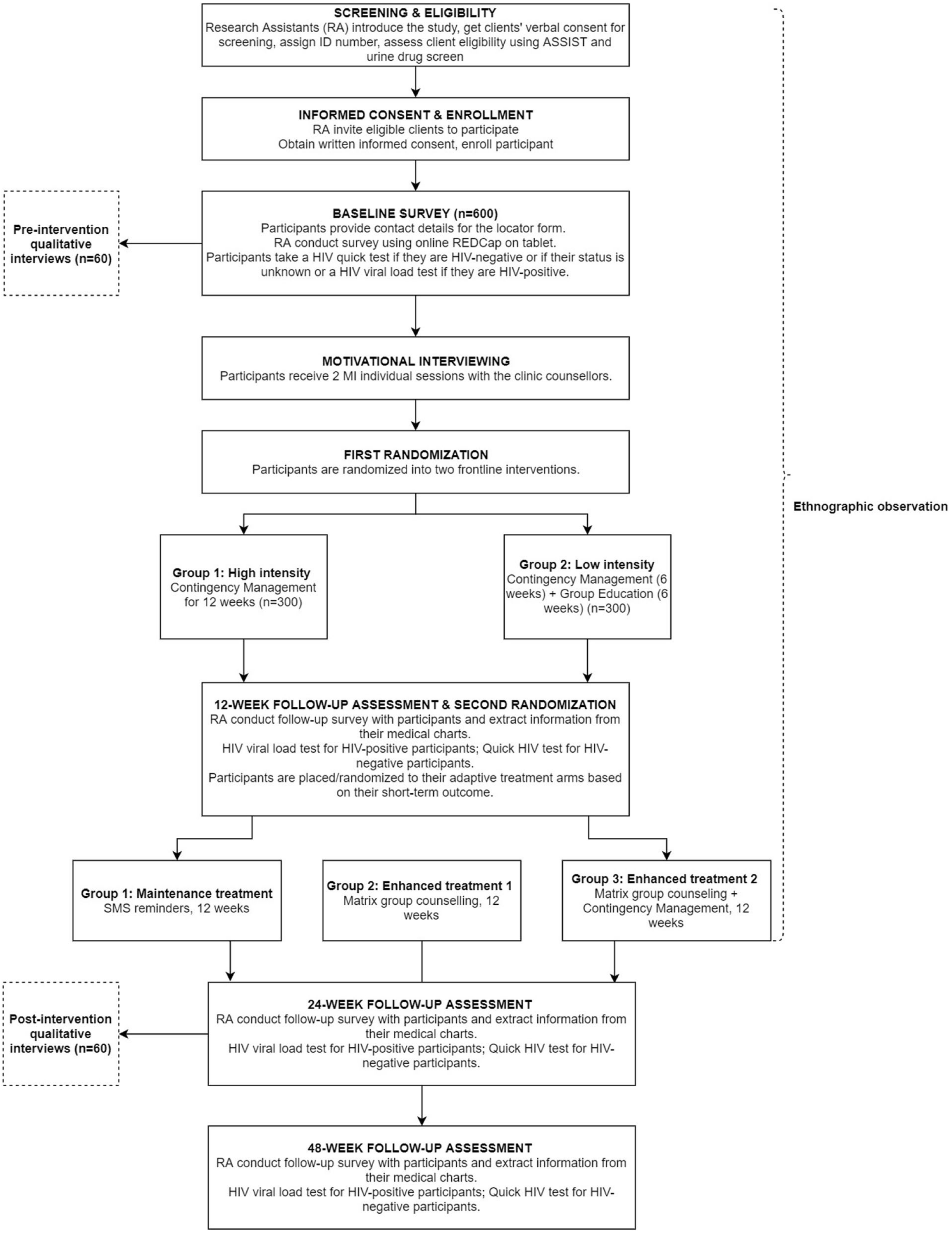

Fig. 1 Study flowchart

interfering problems and (2) inability to understand study procedures by research assistants' judgment. While World Health Organization recommends the ASSIST score of 4 or more, regardless of UDS, as a cut-off point for interventions, drawing from the results of the pilot phase, we have chosen the cut-off point of ASSIST score 10 or more to ensure enough participants move into the adaptive phase.

Methadone providers

Methadone providers will conduct the study interventions under the clinical supervision of the study master counselors. All methadone providers have been trained on basic addiction medicine and will receive further training to deliver the study interventions. We will conduct in-depth interviews with them before and after the intervention phase.

Randomization and interventions

An investigator will stratify participants by HIV status and randomize them into two frontline interventions using REDCap, as shown in Fig. 1. She will send the allocation results to the site research assistants who will then inform providers at the study clinics. This is an open-label study so unblinding does not occur. Participants and their counselors, research assistants, and data managers are aware of participants' assignment of intervention. Participants will receive two individual sessions of motivational interviewing with their counselors before they get into the frontline interventions and, in week 13, before the adaptive strategies start. These sessions would boost participants' motivation for intervention and provide them with greater details of the upcoming intervention activities.

Two frontline interventions (weeks 1–12)

- High intensity: Participants in this arm receive contingency management with the escalating-and-reset schedule throughout 12 weeks [54]. This schedule means participants receive increasing rewards for consecutive negative UDS but if the streak of negative UDS is broken, their rewards return to the starting level. The maximal reward value over 12 weeks is $150 USD, equivalent to the average monthly income of our participants.
- Low intensity: Participants in this arm receive contingency management for the first 6 weeks with the maximal reward value of $40 USD. For the last 6 weeks, they attend weekly group education sessions. The topics of group education include (1) Addiction mechanism, (2) Road to recovery, (3) Coping with triggers, (4) Boredom, (5) Building trust, and (6) Relapse prevention.

Participants with four consecutive UDS negative with methamphetamine in weeks 11 and 12 are considered to be responsive to frontline interventions. Others are considered non-responsive. Responders to frontline interventions are placed in the maintenance treatment arm. Non-responders are randomized to either enhanced treatment 1 or 2.

Three adaptive strategies (weeks 14–25)

- Maintenance treatment: Participants receive two daily automatic unidirectional scripted SMS reminders plus one weekly self-monitoring assessment message over 12 weeks.
- Enhanced treatment 1: Participants attend 12 weekly Matrix group counseling sessions facilitated by the clinic counselors. Our Matrix intervention has the same structure, albeit it is shorter than the original 24-session model [33].
- Enhanced treatment 2: Participants receive the same Matrix intervention plus contingency management over 12 weeks.

Definition of effectiveness measures

Effectiveness measures will be assessed at 13, 26, and 49 weeks after the first week of frontline interventions and throughout the interventions. Primary outcomes include the (1) increase in HIV viral suppression for HIV-positive participants at 26 and 49 weeks, (2) reduction in HIV risk behaviors for both HIV-positive and HIV-negative participants at 26 and 49 weeks, and (3) reduction in methamphetamine use at 13, 26, and 49 weeks measured by UDS (point abstinence) and self-report (continuous abstinence and longest period). Secondary outcomes include (1) adherence to antiretroviral treatment for HIV-positive participants, (2) frequency of HIV testing for HIV-negative participants, (3) heroin use with UDS, (4) opioid overdose, and (5) quality of life.

To acquire the primary and secondary outcomes, we will assess for the following:

- *HIV viral load test*: At baseline, week 13, week 26, and week 49, we send blood samples of HIV-positive participants to laboratories at Bach Mai Hospital in Hanoi and HCMC University of Medicine and Pharmacy Hospital in HCMC for viral load quantification.
- *Drug screening*: We use an FDA-approved Multi-Drug Rapid Test Panel to detect methamphetamine, opioid, and cannabis in urine twice a week throughout the intervention period and at week 49.
- *Methadone and HIV treatment:* We extract methadone and HIV treatment data from participants' medical charts. Variables of interest

include length of treatment, treatment regimen, medication dose, and treatment adherence.

- *Self-reported drug use*: Drug use patterns (methamphetamine, tobacco, and alcohol use) are evaluated based on amphetamine use behaviors, Amphetamine Cessation Symptom Assessment (ACSA), Addiction Severity Index (ASI), and Alcohol Use Disorders Identification Test-Concise (AUDIT-C) scales. ACSA, a 5-point Likert scale, has been used in other studies in Vietnam [55]. The scale includes three domains of methamphetamine withdrawal symptoms: fatigue (3 items), cravings (2 items), and anxiety (11 items) [56]. Higher scores in each domain and in total indicate greater levels of methamphetamine withdrawal symptoms. AUDIT-C is used for identifying patients who drink at hazardous levels or have active alcohol use disorders. AUDIT-C comprises 3 questions scoring on a scale of 0–12 with a cut-off point of 4 for men and of 3 for women [57].
- *Self-reported psychosocial factors*: We use the Barriers to Access to Care Evaluation (BACE) scale to assess barriers to access to mental health care [58]. Participants are asked to what extend they agree or disagree with statements indicating barriers to mental health care. We use the 21-item Depression, Anxiety, Stress Scale (DASS-21) which was previously validated in Vietnam to measure mental health [59, 60]. The DASS-21 scale consists of 7 Likert items for each dimension (depression, anxiety, and stress). For each item, respondents indicate how often it applies to them over the past week. The total score of each dimension will indicate the severity level of depression, anxiety, and stress. The Medical Outcome Study: Social Support Survey scale (MOS-SSS), also validated in Vietnam, is used to measure social support [61, 62]. The MOS-SSS comprises 19 items covering four domains: emotional/informational support, tangible social support, positive social interaction, and affectionate support. Higher scores suggest greater support received. Quality of life is measured with the EQ-5D-5L scale which includes the EQ-5D-5L descriptive system and the EQ Visual Analogue scale (VAS). The descriptive system covers 5 dimensions (mobility, self-care, usual activities, pain/discomfort, anxiety/depression). On the EQ VAS, respondents indicate the score that represents their health status on the scale that ranges from 0 (the worst health they can image) to 100 (the best health they can image). The EQ-5D-5L was previously validated in Vietnam [63].

Economic evaluation

Definition of costs and data collection

We will conduct activity-based costing for each intervention arm over the 20 methadone clinics. A template developed by UNAIDS is adapted to collect data on salaries for personnel and consultants, physical resources, clinical supplies, and miscellaneous charges necessary to deliver each intervention type [64]. Furthermore, any out-of-pocket or indirect costs to the participant will be collected at baseline and at 13 and 26 weeks.

Definition of cost-effectiveness measures

Measures of cost-effectiveness analysis corresponded to the outcomes of interest in Aim 1 including (1) substance use, (2) HIV risk behaviors among HIV-negative participants and HIV viral load, HIV adherence among HIV-positive participants, and (3) Quality of life (Table 1). The cost-effectiveness analysis will measure the increment in cost between contrasted interventions divided by the increment in effectiveness measures.

Ethnographic evaluation

Pre-post intervention in-depth interviews

In each cluster, we will interview 12 key informants including methadone providers, clinic managers, and participants under methadone treatment participating in the study. We will select at minimum 6 participants under treatment so that include old and young, employed and unemployed, and both responsive and non-responsive participants. All participants will receive VND 200,000 (~$10 USD) for their time in each interview. Interviews will be audio-recorded and transcribed verbatim.

Ethnographic observation

This activity is composed of two elements. The first element involves ethnographers spending time with participants with their consent in intervention sessions and other daily activities in the clinic. Such observations will build a rich picture of interventions and intervention settings, including interactions between various groups (intervention providers, other staff, and participants). The second element involves the study master counselors to observe random intervention sessions and assess the fidelity of intervention delivery using a checklist.

Training and fidelity monitoring

In each selected clinic, a physician, two counselors, and one nurse will participate in the study as intervention providers. The physician will ensure referral to HIV and psychiatric services when necessary; two counselors will run motivational interviewing, group education sessions, and Matrix meetings; the nurse will collect urine twice a week and conduct contingency management based on the UDS results. Before the start of the intervention, to ensure the accuracy,

Table 1 Assessment schedule

Assessments	*Baseline*	*Week 13*	*Week 26*	*Week 49*
**Sociodemographic characteristics**	X			X
**Drug use history**	X		X	X
**Current drug use**	X		X	X
Amphetamine use behaviors				
Amphetamine Cessation Symptom Assessment (ACSA)				
**Tobacco smoking**	X		X	X
**Alcohol use** (AUDIT-C)	X		X	X
**Barriers to access to care**	X		X	X
BACE				
**Mental health**	X		X	X
Depression, Anxiety, Stress Scale (DASS-21)				
**Social support (MOS-SS**)	X		X	X
**Quality of life (EQ-5D-5L)**	X			X
**Stigmatization**	X			X
Towards people who are under methadone treatment (MMT-SMS)				
Toward people who use drugs				
Toward people living with HIV				
**Treatment information (from medical charts)**	X	X	X	X
Methadone maintenance treatment				
HIV treatment				
**Cost data**	X		X	X
Opportunity costs				
Other healthcare costs				
Social costs				
**Tests**				
Quick and confirmatory HIV tests (not for participants with known HIV seropositivity)	X	X	X	X
Viral load test	X	X	X	X
UDS	Twice a week throughout intervention			X
**Ethnographic observation**	X	X	X	X
**Qualitative interviews**	X		X	

integrity, and fidelity to the EBIs, all intervention staff at methadone clinics will (1) receive didactic training on the theory behind the approach, (2) evaluate their comprehension of the concepts within and behind the approach, (3) watch a video of a Master Behavioral Counselor conducting intervention sessions and discuss the details of the session, and (4) conduct at least two pilot intervention instances. All intervention sessions, except contingency management, will be audio-recorded, transcribed, and coded to ensure intervention fidelity. Intervention staff who have lower levels of intervention integrity or who have significant drift will be provided detailed feedback and supervision until there is parity with other staff.

Sample size determination

Sample sizes were chosen to compare primary outcomes based on first-stage randomization into one of two groups: high intensity or low intensity frontline interventions. Sample size calculations are conducted in PASS 2008 [65] for a two-group comparison of binary outcomes, a power of 80%, a 5% alpha level, and a conservative attrition rate of 20%. Using estimates from our prior work, we anticipate base rates of 80 to 90% for substance use and 60 to 70% for viral suppression. Based on these assumptions and a proposed sample of 200 HIV-positive participants (with 100 participants per group), we can detect randomization group differences of 20% or more for binary outcomes, such as substance use and viral

load suppression. We can detect even smaller group differences for substance use outcomes in the proposed sample of 400 HIV-negative participants and the combined sample of HIV-positive and HIV-negative participants. If estimated outcome probabilities are similar between first-stage randomization groups at 12 weeks, we will pool 12-week results for even greater power in evaluating second-stage randomization differences.

Data management

Different datasets collected from different sources will be linked through a unique identification code using REDCap (Research Electronic Data Capture) for quantitative data. Data will be uploaded in real-time from the 20 study clinics onto our database. The study data manager will assess transferred data for completeness, query sites regarding any inconsistencies, and code merged data files for analysis.

For qualitative data, field notes written on site are expanded and recorded electronically within 24 h. After removing all personal identifiable information, the research team will upload password-protected transcripts on a secured database. The transcripts will be uploaded into Atlas.ti software to organize data and facilitate analysis.

Data analysis

To assess effectiveness

We will use a time-varying mixed-effects model that will be fitted to the participants' common outcome measures over time [66]. The unadjusted model will include indicators of first-stage and second-stage intervention conditions, time of the assessment (baseline, 13, 26, and 49 weeks), and intervention indicators-by-time interaction terms. An additional interaction term of the two intervention indicators will be included to account for any interaction effect between the first and the second stage interventions. The adjusted model will include patients' socio-demographic characteristics, drug use history, HIV-serostatus, and location as fixed effects. The mixed-effects models will include a participant-level random effect to account for repeated observations of each participant, as well as a clinic-level random effect to account for the nested nature within the clinics.

We will conduct subgroup analyses among HIV-positive and HIV-negative participants. For the HIV-positive subgroup, the specific outcomes of interest include (1) HIV viral load suppression and (2) adherence to antiretroviral treatment, and specific outcomes for HIV-negative subgroup include (1) frequency of HIV testing and (2) HIV seroconversion. Substance use will be the common outcomes in models including participants of both HIV statuses.

To assess cost-effectiveness

We will calculate cost-effectiveness ratios (CER) for each of the intermediate and final outcomes. The CER is in the broadest terms the difference in per capita costs of administering one intervention ($C1 - C2$) relative to a second, divided by the difference in outcomes between the two interventions ($O1 - O2$):

$$\mathrm{CER} = \frac{C1 - C2}{O1 - O2}$$

For example, calculating the CER for adding contingency management to Matrix for non-responders would yield a CER equation. In calculating the CER of high vs. low intensity contingency management at the first randomization stage, the entire range of subsequent costs will be included. Costs of delivering the interventions will be derived from clinic records of time and other inputs, as well as incentive payments, thus providing an estimate of CER from the medical system perspective. We will also evaluate CERs from a societal perspective, using a broad definition of costs, including the social costs of incarceration.

We will conduct sensitivity analyses [67] to estimate the extent to which the CER calculation is affected by differences in assumptions about the size of the differences in intervention effect. In particular, we will determine how sensitive the CER is to assumptions that the difference in treatment effect is one standard deviation below or above the mean estimated effect size. Similarly, we will estimate the sensitivity of conclusions to costs that are one standard deviation below or above the estimated mean.

To identify the factors influencing the adoption and scale up of the model

The qualitative analysis team will read and provide a narrative summary for each transcript. A codebook will be developed based on these summaries. Memo-writing and code-refining will be conducted throughout the analysis. Iterative analyses assess convergence of patient, provider and organizational dimensions on study measures, and the context of the policy subsystems, cross system interactions, and resource allocation.

Analysis of non-adherence and missing data

We will first describe the extent and patterns of missingness within each variable and check for associations between missing and observed data to determine the mechanism of missingness, which could be missing completely at random, missing at random, or missing not at random. Missing data will then be handled using multiple imputation [68]. Appropriate imputation techniques will be chosen for the type of missing data and

the statistical tools employed [69]. For sensitivity analysis, we will conduct analyses with and without multiple imputations. All participants will be analyzed on an intent-to-treat basis where the study outcomes are examined based on the random intervention assignment and not on the actual intervention received or adherence to the intervention [70].

Interim analyses

There is no planned interim analysis as the behavioral therapies used in this trial have no known serious adverse events and are consistently more efficacious than control conditions in treatment-seeking participants [71]. The effect sizes of the behavioral therapies in this trial are in the moderate range [71]. Furthermore, any interim analysis and decision to stop the trial would likely be based on underpowered data and susceptible to error.

Oversight and monitoring

Scientific advisory committee

The study scientific advisory committee members include researchers, policy makers, and activists working in HIV and addiction medicine fields in the USA and Vietnam. The scientific advisory committee meets once a year to review research progress and key findings, as well as discuss challenges to study implementation and plans to solve these challenges.

Data monitoring committee

Our data monitoring committee is composed of members of the Data and Safety Monitoring Board for Addiction Medicine (DSMBAM) of the University of California – Los Angeles. These members are not connected to the study in any way. The DSMBAM is independent from the National Institute on Drug Abuse (NIDA)—the sponsor of this study. The DSMBAM meets quarterly to monitor subjects' progress in the trial and considers whether adverse social harms (e.g., police detention, hospitalization due to overdose) differentially accrue by condition. Although there are no prospective stopping rules for this trial, the DSMBAM is within its charge to review aggregate data, request statistical tests of differences in social or other harms, and then advise changes in intervention type or intensity if statistically significant differences emerge in adverse events by condition. Prior to each meeting, the study team will submit a performance report including all reports of SAEs for DSMBAM's consideration. After each meeting, recommendations will be made in writing to the principal investigators.

Auditing trial conduct

Hanoi Medical University and the staff in the STAR-OM study provide oversight of financial management. The Vietnam teams and US teams maintain frequent communication via emails and bi-weekly online meetings to report updates on the study progress, discuss scientific aspects of the study, and troubleshoot issues when they arise. The teams in Hanoi and HCMC meet online once weekly and in-person quarterly during monitoring visits to discuss the study conduct. We submit annual research progress reports to the Ethics Committee of Hanoi Medical University. Any protocol amendments need to get ethical approval before implementation. The UCLA Addiction Medicine Data Safety Monitoring Board independently review our data and data management twice a year.

Adverse event reporting and harms

Adverse events in this trial are defined as medical issues that do not require hospitalization. Serious adverse events are defined as life-threatening events such (e.g., suicide, opioid overdose) or other events that have a negative impact on participants' life such as incarceration or compulsory drug rehabilitation. The clinic staff will communicate information about adverse events and serious adverse events to the study team right after they are informed by participants or participant families. The study coordinators in Hanoi and HCMC are responsible to report adverse events within 7 days and serious adverse events within 24 h on REDCap with the time of onset, seriousness, duration, and outcomes. The principal investigator will decide what serious adverse events need to be reported to the Ethics Committee.

Plan for communicating important protocol amendment to relevant parties

We will first seek advice of our scientific advisory committee for all protocol amendments. Protocol amendments will undergo the review of Ethics Committee. If they are approved, we will notify the trial funder about these amendments. No protocol modifications will be implemented without ethical approval. Notification of the approved modifications will be forwarded to all study team members.

Dissemination plans

Results of this study will be published in peer-reviewed scientific articles and presented at international and regional conferences. We will organize dissemination workshops to communicate the study results to policy makers and healthcare professionals at the end of the trial. The de-identified datasets, statistical code, and full protocol are available from the first author upon reasonable request.

Ethics and confidentiality

Prior to participation in the trial, the participant will be informed about the research. Participants will complete a short questionnaire about the study objectives and main activities to show how they understand the study. Research assistants will provide more explanation based on the results of the questionnaire. If participants agree to join the study, they will sign a consent form. Each participant will be assigned a unique identifier at the time of screening. Participant data will be linked to this identifier only. Participant personal identifiable information is stored in a separate locked cabinet to which only responsible study staff have access. All study staff sign a confidentiality agreement to non-disclosure of participant information. We make extra efforts to ensure no-disclosure of drug use information to anyone other than participants and the study staff.

Provision of post-trial care

Participants continue to receive usual methadone treatment at post-trial. While there is no formal provision of methamphetamine intervention after the trial completes, the clinic staff with experiences in methamphetamine intervention would likely provide better service for participants in need.

Challenges and adaptations

Intervention adaptation

Between July and October 2020, we conducted 4 focus group discussions (FGD) of a convenience sample of participants from four methadone clinics in the downtown and suburbs of Hanoi and HCMC to inform intervention content and refinement. Respondents reported information on local taxonomy and patterns of methamphetamine use, triggering situations, methamphetamine-related sexual risks, motivations for seeking treatment, and perceived acceptability of the adaptive interventions.

The pilot implementation lasted 12 weeks from November 2020 through February 2021. It identified issues to be addressed before the full implementation. At the conclusion of the pilot, we conducted 2 FGD with patients and 1 FGD with providers participating in the pilot to gauge their feedback about the interventions.

Challenges and modifications

Modification of eligibility criteria With the cut-off point of ASSIST ≥ 4 and methamphetamine-positive UDS as originally proposed, there were 26 and 52 eligible participants in two pilot clinics in Hanoi and HCMC, respectively (see Table 2). For the pilot implementation, we randomly recruited 42 participants with ASSIST score ≥ 4 or methamphetamine-positive UDS. After the frontline intervention, 16 (38%) participants were non-responders and randomized into adaptive interventions. At least 50% of the original sample must transition to the adaptive phase for sufficient statistical power. Thus, we decided to recruit more participants with severe use of methamphetamine, as evidenced in both ASSIST score ≥ 10 and methamphetamine-positive UDS. Furthermore, to recruit enough participants for the frontline intervention phase, given most other clinics are smaller than the two pilot ones, we decided to use ASSIST score "OR" UDS instead of "AND" to increase the pool of potential participants. We kept the criterion of methamphetamine-positive UDS to compensate for participants with lower ASSIST scores due to desirability bias.

Modification to minimize the impacts of the COVID-19 pandemic Since late April 2021, the COVID-19 epidemic in Vietnam was severe. HCMC applied a strict lockdown from early July 2021 through September 2021. Some methadone clinics were temporarily closed due to confirmed COVID-19 cases among patients; medications were delivered at community-based healthcare centers. In the clinics that remained open, clinical activities other than medication dispensing ceased to minimize contact between providers and patients. Unlike HCMC, clinical activities in Hanoi continued, albeit at levels lower than pre-pandemic. In addition, methadone clinics in both cities suffered from staff shortages as many staff were deployed to support ongoing COVID-19 prevention and treatment activities. With advice of the study's Scientific Advisory Board, we developed a response plan to potential COVID-19 interruptions to minimize the pandemic's impact (see Fig. 2).

For intervention:

- For group education and Matrix sessions, we will conduct small groups of five or fewer people during the surge of the COVID-19 pandemic when the city authorities forbid large meetings. If we cannot conduct group sessions, we will provide individual sessions of the same content to ensure all participants receive interventions.

Table 2 Number of eligible participants by original criteria

	# eligible (ASSIST + UDS)	UDS (+)	ASSIST ≥ 4
Hanoi clinic (*N* = 350)	26	31	114
HCMC clinic (*N* = 385)	52	64	122

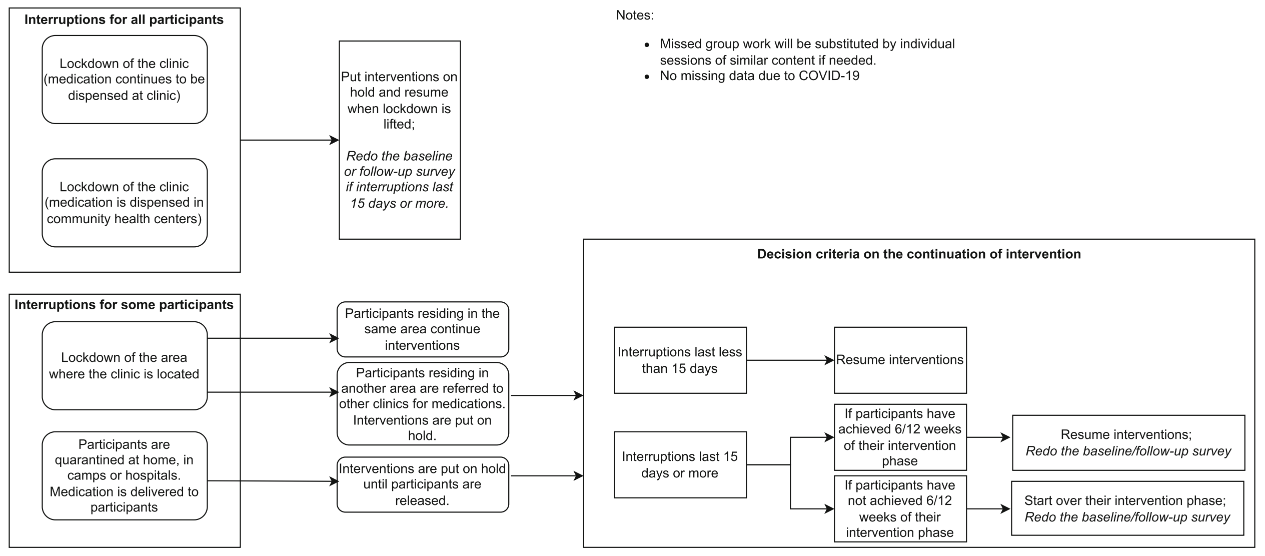

Fig. 2 Response plan to COVID-19-related interruptions

- For participants who miss scheduled visits due to COVID-19, we will consider whether to resume the intervention where they left off or to restart their intervention phase. This will depend upon (1) the length of the interruption (15 days or less) and (2) whether participants have gone through 50% of their scheduled intervention sessions before the interruption.

Discussion

The STAR-OM study is among the first studies to evaluate different combinations of EBIs for methamphetamine use among methadone patients in low-and-middle-income countries. The study will provide effectiveness and cost-effectiveness evidence for scaling up these interventions. The SMART design assesses different treatment strategies for participants who respond differently to frontline interventions. The combination of trial and ethnographical study will provide insights on factors at multiple levels that need to be considered in decision-making. The adaptation and pilot implementation of EBIs will make them culturally sound to local participants. As the interventions will be delivered by methadone providers at methadone clinics, they can be readily implemented if the trial demonstrates they help.

The participation of some participants can be interrupted due to drug-related police arrest or methadone treatment fatigue. This limitation can be minimized as we will select clinics with low drop-out rates. We have officially informed the local police on the study implementation and received approval from both national and local authorities. While this measure does not prevent participants from being arrested, especially when they are involved in illegal activities, it could reduce attrition. Furthermore, the COVID-19 pandemic and containment measures could pose challenges for the study implementation. With the response plan developed for potential interruption scenarios, we believe the study will be implemented safely and will maintain a high-level of data quality and intervention fidelity.

Conclusion

The findings of this study may greatly contribute to the implementation of EBIs for methamphetamine use among methadone patients in Vietnam and other low-resourced settings. The effectiveness and cost-effectiveness findings will be critical to make decisions on adopting methamphetamine use interventions. The study will provide insights into the barriers and facilitators to the expansion of interventions to direct further policy advocacy and program development. The study will be implemented at the time when the COVID-19 pandemic has waged significant impacts in Vietnam. Therefore, it offers lessons learned for future scale-up of the intervention in the face of the continuing COVID-19 pandemic in many parts of the world.

Trial status

Protocol version 2.0, dated 25 March 2021

Recruitment start date: 14 June 2021

Estimate recruitment completion date: 31 March 2024

Acknowledgements

We thank our research assistants for their help in the data collection. We are grateful to the methadone practitioners and patients for their participation in our study. The study's advisory board provided us with valuable advices on how to deal with the possible implementation interruptions due to COVID-19 pandemic.

Organizational structure and responsibilities
Principal Investigators: Le Minh Giang and Steve Shoptaw
Design and conduct of STAR-OM
Preparation of protocol and revisions
Organizing scientific advisory board meetings
Publication of study reports
Co-Investigators: Hoang Thi Hai Van, Nguyen Van Tuan, Do Van Dung, and Thai Thanh Truc
Development of the study standards of operation and data collection tools
Supervising the data collection and analysis process
Development of manuscripts
Steering committee: Gavin Bart, Don Desjarlais, Khuat Thi Hai Oanh, Hoang Dinh Canh, and Richard Rawson
Agreement of final protocol
Reviewing progress of study and if necessary agreeing changes to the protocol to facilitate the smooth running of the study
Data Manager: Nguyen Bich Diep
Maintenance of trial IT system and data entry
Data verification and analysis
Project Director: Nguyen Thu Trang
Overseeing the study implementation
Preparation of study reports
Project Director Assistant: Vu Thi Tuong Vi
Overseeing the study implementation in Ho Chi Minh City
Ethnographer: Dinh Thi Thanh Thuy and Nguyen Hong Hoa
Development of qualitative data collection tools
Conducting in-depth interviews, focus group discussion and field observation
Intervention Coaches and Master Behavioural Counselors: Han Dinh Hoe, Nguyen Huu Anh, Nguyen Ly Lai, and Pham Thi Dan Linh
Providing training and technical assistance to practitioners at methadone clinics who deliver the study interventions
Providing the SMS messaging intervention
Data and Safety Monitoring Board for Addiction Medicine of the University of California, Los Angeles (UCLA DSMBAM)—composition and roles
The practices of UCLA DSMBAM indicate that the exact list of subcommittee members who review a study is not provided to keep them somewhat anonymous. Each committee consists of 3–5 members.
Below are members who serve as committee chairs as well as clinical research experts:

- *Timothy M. Hall, MD, PhD,* is Health Sciences Assistant Clinical Professor at UCLA Department of Family Medicine and Center for Behavioral and Addiction Medicine. His research expertise is in sexual identity formation, social/sexual MSM networks, non-gay-identified MSM, ethnography, and MSM substance use.
- *Gayle Baldwin, PhD,* is Associate Professor in the Department of Medicine at the University of California, Los Angeles. Her research focuses on how specific immune cells fight infectious diseases and cancer.
- *Dominick Frosch, PhD,* is an Associate Staff Scientist in the Department of Health Services Research at the Palo Alto Medical Foundation's Research Institute. He is also an Assistant Professor of Medicine at the University of California, Los Angeles. His research is focused on developing and implementing interventions to increase patient participation in clinical decision making and understanding the effects of health information in the media on behavior.
- *Liz Evans, PhD, MA,* is an Associate Professor in the Department of Health Promotion and Policy at the University of Massachusetts Amherst School of Public Health and Health Sciences. Her research is focused on how health care systems and public policies can better promote health and wellness among vulnerable and underserved populations, particularly for individuals at risk for substance use disorders, mental illness, and infectious diseases.
- *Timothy W. Fong, MD,* is a Clinical Professor of Psychiatry at the UCLA Jane and Terry Semel Institute for Neuroscience and Human Behavior. He co-directs a program aimed at examining the underlying causes and clinical characteristics of gambling disorder in order to develop effective, evidence-based treatment strategies. He is also involved in research to examine the full impact of cannabis on the body, mind, and brain.

Below are members who serve as experts in biostatistics and epidemiology:

- Jesse Fletcher, PhD
- Sung-Jae Lee, PhD
- Glenn-Milo Santos, MPH, PhD
- Robert Weiss, PhD

Below are members who serve as clinical research experts:

- Adam Carrico, PhD
- Jesse L. Clark, MD
- Phillip O. Coffin, MD, MIA
- David Goodman-Meza, MD, MAS
- Emeka Okafor, PhD
- Lara Ray, PhD
- Sae Takada, MD, PhD, MS

Administrative support staff:

- Uyen Kao, MPH, Director
- Oluwadamilola O. Jolayemi, MSc, Coordinator

Authors' contributions
LMG and SS designed and lead the study. DVD leads the study in HCMC site. NTT oversees the implementation in the two study sites and led the writing of the manuscript. NBD analyzed the study pilot data. DTDT and AL wrote the section on economic evaluation. HDH, NLL, and PTDL revised the intervention strategies with inputs from the pilot implementation. TTT, HHN, CR, CL, and ML contributed to the revision of the manuscript. All authors read and approved the final manuscript.

Funding
The National Institute on Drug Abuse is the funder and sponsor of this study (1R01DA050486-01). This funding source had no role in the design of this study and will not have any role during its execution, analyses, interpretation of the data, or decision to submit results.
Name and contact of the sponsor: https://nida.nih.gov/about-nida/contact-us
Office of Science Policy and Communications, National Institute on Drug Abuse, 3WFN MSC 6024, 301 North Stonestreet Ave, Bethesda, MD 20892, 301-443-1124 (General Inquiries), 301-443-6245 (Media Inquiries)

Availability of data and materials
Not applicable

Declarations

Ethics approval and consent to participate
Ethics approval has been obtained through Hanoi Medical University (#122/GCNHDDDNCYSH-ĐHYHN) and University of Medicine and Pharmacy in HCMC, Vietnam (#433/HDDD-ĐHYD).

Consent for publication
Not applicable

Competing interests
The authors declare that they have no competing interests

Author details
[1]Centre for Training and Research on Substance use and HIV, Hanoi Medical University, Hanoi, Vietnam. [2]Global Health Department, Hanoi Medical University, Hanoi, Vietnam. [3]Faculty of Public Health, University of Medicine and Pharmacy at Ho Chi Minh City, Ho Chi Minh City, Vietnam. [4]University of Medicine and Pharmacy at Ho Chi Minh City, Ho Chi Minh City, Vietnam. [5]South Vietnam HIV and Addiction Technology Transfer Center, University of Medicine and Pharmacy at Ho Chi Minh City, Ho Chi Minh City, Vietnam. [6]Friends Research Institute, Friends Community Center, Center for HIV Identification, Prevention and Treatment Services, University of California, Los Angeles, Los Angeles, USA. [7]Department of Public Policy, Luskin School of Public Affairs, University of California, Los Angeles, Los Angeles, USA. [8]Department of Epidemiology, Fielding School of Public Health, University of

California, Los Angeles, Los Angeles, USA. [9]Department of Psychiatry and Biobehavioral Sciences, Jane & Terry Semel Institute for Neuroscience & Human Behavior, University of California, Los Angeles, Los Angeles, USA. [10]Center for Behavioral and Addiction Medicine, Department of Family Medicine, University of California, Los Angeles, Los Angeles, USA.

Received: 21 October 2021 Accepted: 4 April 2022
Published online: 23 April 2022

References

1. Ellis MS, Kasper ZA, Cicero TJ. Twin epidemics: the surging rise of methamphetamine use in chronic opioid users. Drug Alcohol Depend. 2018;193:14–20. https://doi.org/10.1016/j.drugalcdep.2018.08.029.
2. Giang L, Thuy D, Trang N, Okafor C, Li M, Shoptaw S. Severity of methamphetamine use among methadone patients in Vietnam: prevalence and correlates. San Antonio; 2019.
3. Michel L, Des Jarlais DC, Duong Thi H, Khuat Thi Hai O, Pham Minh K, Peries M, et al. Intravenous heroin use in Haiphong, Vietnam: need for comprehensive care including methamphetamine use-related interventions. Drug Alcohol Depend. 2017;179:198–204. https://doi.org/10.1016/j.drugalcdep.2017.07.004.
4. UNODC GS programme. Synthetic drugs in East and Southeast Asia - latest developments and challenges; 2020. p. 116.
5. Bao Y-P, Liu Z-M, Li J-H, Zhang R-M, Hao W, Zhao M, et al. Club drug use and associated high-risk sexual behaviour in six provinces in China. Addiction. 2015;110(Suppl 1):11–9. https://doi.org/10.1111/add.12770.
6. Carrico AW, Nil E, Sophal C, Stein E, Sokunny M, Yuthea N, et al. Behavioral interventions for Cambodian female entertainment and sex workers who use amphetamine-type stimulants. J Behav Med. 2016;39(3):502–10. https://doi.org/10.1007/s10865-016-9713-2.
7. Colfax G, Santos G-M, Chu P, Vittinghoff E, Pluddemann A, Kumar S, et al. Amphetamine-group substances and HIV. Lancet Lond Engl. 2010;376(9739):458–74. https://doi.org/10.1016/S0140-6736(10)60753-2.
8. Zhang C, Liu Y, Sun X, Wang J, Lu H-Y, He X, et al. Substance use and HIV-risk behaviors among HIV-positive men who have sex with men in China: repeated measures in a cohort study design. AIDS Care. 2017;29(5):644–53. https://doi.org/10.1080/09540121.2016.1255709.
9. Liu D, Gu J, Xu H, Hao C, Jiao M, Zhang X, et al. Club drugs and alcohol abuse predicted dropout and poor adherence among methadone maintenance treatment patients in Guangzhou, China. AIDS Care. 2017;29(4):458–63. https://doi.org/10.1080/09540121.2016.1259452.
10. Wang R, Ding Y, Bai H, Duan S, Ye R, Yang Y, et al. Illicit heroin and methamphetamine use among methadone maintenance treatment patients in Dehong Prefecture of Yunnan Province, China. PLoS One. 2015;10(7):e0133431. https://doi.org/10.1371/journal.pone.0133431.
11. Carrico AW, Cherenack EM, Roach ME, Riley ED, Oni O, Dilworth SE, et al. Substance-associated elevations in monocyte activation among methamphetamine users with treated HIV infection. AIDS Lond Engl. 2018;32(6):767–71. https://doi.org/10.1097/QAD.0000000000001751.
12. Carrico AW, Hunt PW, Neilands TB, Dilworth SE, Martin JN, Deeks SG, et al. Stimulant use and viral suppression in the era of universal antiretroviral therapy. J Acquir Immune Defic Syndr. 2019;80(1):89–93. https://doi.org/10.1097/QAI.0000000000001867.
13. Carrico AW, Flentje A, Kober K, Lee S, Hunt P, Riley ED, et al. Recent stimulant use and leukocyte gene expression in methamphetamine users with treated HIV infection. Brain Behav Immun. 2018;71:108–15. https://doi.org/10.1016/j.bbi.2018.04.004.
14. Horvath KJ, Carrico AW, Simoni J, Boyer EW, Amico KR, Petroll AE. Engagement in HIV medical care and technology use among stimulant-using and nonstimulant-using men who have sex with men. AIDS Res Treat. 2013;2013:121352–11. https://doi.org/10.1155/2013/121352.
15. Korthuis P, Todd B, Gavin KL, Diep NB, Brinkerhoff C, Giang LM. Buprenorphine versus Methadone impact on the HIV care continuum in Vietnam: BRAVO study 6-month outcomes. San Diego; 2018.
16. Alam-mehrjerdi Z, Mokri A, Dolan K. Methamphetamine use and treatment in Iran: a systematic review from the most populated Persian Gulf country. Asian J Psychiatr. 2015;16:17–25. https://doi.org/10.1016/j.ajp.2015.05.036.
17. Shariatirad S, Maarefvand M, Ekhtiari H. Methamphetamine use and methadone maintenance treatment: an emerging problem in the drug addiction treatment network in Iran. Int J Drug Policy. 2013;24(6):e115–6. https://doi.org/10.1016/j.drugpo.2013.05.003.
18. Watt MH, Meade CS, Kimani S, MacFarlane JC, Choi KW, Skinner D, et al. The impact of methamphetamine ("tik") on a peri-urban community in Cape Town, South Africa. Int J Drug Policy. 2014;25(2):219–25. https://doi.org/10.1016/j.drugpo.2013.10.007.
19. Baingana F. al'Absi M, Becker AE, Pringle B. Global research challenges and opportunities for mental health and substance-use disorders. Nature. 2015;527(7578):S172–7. https://doi.org/10.1038/nature16032.
20. Petersen I, Evans-Lacko S, Semrau M, Barry MM, Chisholm D, Gronholm P, et al. Promotion, prevention and protection: interventions at the population- and community-levels for mental, neurological and substance use disorders in low- and middle-income countries. Int J Ment Heal Syst. 2016;10(1):30. https://doi.org/10.1186/s13033-016-0060-z.
21. AshaRani P, Hombali A, Seow E, Ong WJ, Tan JH, Subramaniam M. Non-pharmacological interventions for methamphetamine use disorder: a systematic review. Drug Alcohol Depend. 2020;212:108060. https://doi.org/10.1016/j.drugalcdep.2020.108060.
22. Knight R, Karamouzian M, Carson A, Edward J, Carrieri P, Shoveller J, et al. Interventions to address substance use and sexual risk among gay, bisexual and other men who have sex with men who use methamphetamine: a systematic review. Drug Alcohol Depend. 2019;194:410–29. https://doi.org/10.1016/j.drugalcdep.2018.09.023.
23. Radfar SR, Rawson RA. Current research on methamphetamine: epidemiology, medical and psychiatric effects, treatment, and harm reduction efforts. Addict Health. 2014;6(3–4):146–54.
24. Polcin DL, Bond J, Korcha R, Nayak MB, Galloway GP, Evans K. Randomized trial of intensive motivational interviewing for methamphetamine dependence. J Addict Dis. 2014;33(3):253–65. https://doi.org/10.1080/10550887.2014.950029.
25. Galloway GP, Polcin D, Kielstein A, Brown M, Mendelson J. A nine session manual of motivational enhancement therapy for methamphetamine dependence: adherence and efficacy. J Psychoactive Drugs. 2007;39(sup4):393–400.
26. Benishek LA, Dugosh KL, Kirby KC, Matejkowski J, Clements NT, Seymour BL, et al. Prize-based contingency management for the treatment of substance abusers: a meta-analysis. Addiction. 2014;109(9):1426–36. https://doi.org/10.1111/add.12589.
27. Brown HD, DeFulio A. Contingency management for the treatment of methamphetamine use disorder: a systematic review. Drug Alcohol Depend. 2020;216:108307. https://doi.org/10.1016/j.drugalcdep.2020.108307.
28. Dutra L, Stathopoulou G, Basden SL, Leyro TM, Powers MB, Otto MW. A meta-analytic review of psychosocial interventions for substance use disorders. Am J Psychiatry. 2008;165(2):179–87. https://doi.org/10.1176/appi.ajp.2007.06111851.
29. Griffith JD, Rowan-Szal GA, Roark RR, Simpson DD. Contingency management in outpatient methadone treatment: a meta-analysis. Drug Alcohol Depend. 2000;58(1–2):55–66. https://doi.org/10.1016/S0376-8716(99)00068-X.
30. Prendergast M, Podus D, Finney J, Greenwell L, Roll J. Contingency management for treatment of substance use disorders: a meta-analysis: Centre for Reviews and Dissemination (UK); 2006. [cited 2020 Feb 25]. Available from: https://www.ncbi.nlm.nih.gov/books/NBK72392/
31. Lussier JP, Heil SH, Mongeon JA, Badger GJ, Higgins ST. A meta-analysis of voucher-based reinforcement therapy for substance use disorders. Addiction. 2006;101(2):192–203. https://doi.org/10.1111/j.1360-0443.2006.01311.x.
32. Ginley MK, Pfund RA, Rash CJ, Zajac K. Long-term efficacy of contingency management treatment based on objective indicators of abstinence from illicit substance use up to 1 year following treatment: a meta-analysis. J Consult Clin Psychol. 2021;89(1):58–71. https://doi.org/10.1037/ccp0000552.
33. Rawson RA, Marinelli-Casey P, Anglin MD, Dickow A, Frazier Y, Gallagher C, et al. A multi-site comparison of psychosocial approaches for the treatment of methamphetamine dependence. Addiction. 2004;99(6):708–17. https://doi.org/10.1111/j.1360-0443.2004.00707.x.
34. Reback CJ, Fletcher JB, Shoptaw S, Mansergh G. Exposure to theory-driven text messages is associated with HIV risk reduction among methamphetamine-using men who have sex with men. AIDS Behav. 2015;19(Suppl 2):130–41. https://doi.org/10.1007/s10461-014-0985-7.
35. Reback CJ, Fletcher JB, Swendeman DA, Metzner M. Theory-based text-messaging to reduce methamphetamine use and HIV sexual risk behaviors among men who have sex with men: automated unidirectional delivery

outperforms bidirectional peer interactive delivery. AIDS Behav. 2019;23(1):37–47. https://doi.org/10.1007/s10461-018-2225-z.
36. Reback CJ, Fletcher JB, Kisler KA. Text messaging improves HIV care continuum outcomes among young adult trans women living with HIV: Text Me, Girl! AIDS Behav. 2021;25(9):3011–23. https://doi.org/10.1007/s10461-021-03352-3.
37. Reback CJ, Fletcher JB, Leibowitz AA. Cost effectiveness of text messages to reduce methamphetamine use and HIV sexual risk behaviors among men who have sex with men. J Subst Abus Treat. 2019;100:59–63. https://doi.org/10.1016/j.jsat.2019.02.006.
38. Gómez W, Olem D, Andrews R, Discepola MV, Ambrose P, Dilworth SE, et al. Optimizing contingency management with methamphetamine-using men who have sex with men. Cogn Behav Pract. 2018;25(2):286–95. https://doi.org/10.1016/j.cbpra.2017.08.003.
39. Parsons JT, John SA, Millar BM, Starks TJ. Testing the efficacy of combined motivational interviewing and cognitive behavioral skills training to reduce methamphetamine use and improve HIV medication adherence among HIV-positive gay and bisexual men. AIDS Behav. 2018;22(8):2674–86. https://doi.org/10.1007/s10461-018-2086-5.
40. Dugosh K, Abraham A, Seymour B, McLoyd K, Chalk M, Festinger D. A systematic review on the use of psychosocial interventions in conjunction with medications for the treatment of opioid addiction. J Addict Med. 2016;10(2):91–101. https://doi.org/10.1097/ADM.0000000000000193.
41. Assanangkornchai S, Balthip Q, Edwards JG. assistance of the ASSIST-SBI Development Co-investigators. Implementing the Alcohol, Smoking, Substance Involvement Screening Test and linked brief intervention service in primary care in Thailand. J Public Health Oxf Engl. 2014;36(3):443–9. https://doi.org/10.1093/pubmed/fdu011.
42. Sorsdahl K, Myers B, Ward C, Matzopoulos R, Mtukushe B, Nicol A, et al. Screening and brief interventions for substance use in emergency departments in the Western Cape province of South Africa: views of health care professionals. Int J Inj Control Saf Promot. 2014;21(3):236–43. https://doi.org/10.1080/17457300.2013.811267.
43. Wechsberg WM, Luseno WK, Karg RS, Young S, Rodman N, Myers B, et al. Alcohol, cannabis, and methamphetamine use and other risk behaviours among Black and Coloured South African women: a small randomized trial in the Western Cape. Int J Drug Policy. 2008;19(2):130–9. https://doi.org/10.1016/j.drugpo.2007.11.018.
44. Chen W, Hong Y, Zou X, McLaughlin MM, Xia Y, Ling L. Effectiveness of prize-based contingency management in a methadone maintenance program in China. Drug Alcohol Depend. 2013;133(1):270–4. https://doi.org/10.1016/j.drugalcdep.2013.05.028.
45. Hser Y-I, Li J, Jiang H, Zhang R, Du J, Zhang C, et al. Effects of a randomized contingency management intervention on opiate abstinence and retention in methadone maintenance treatment in China. Addiction. 2011;106(10):1801–9. https://doi.org/10.1111/j.1360-0443.2011.03490.x.
46. Cohen A, McGregor C. The emergence of methamphetamine in Thailand: interventions and treatment. In: Pates R, Riley D, editors. Interventions for Amphetamine Misuse. Oxford: Wiley-Blackwell Publishing; 2010. p. 159–71.
47. Magidson JF, Gouse H, Burnhams W, Wu CYY, Myers B, Joska JA, et al. Beyond methamphetamine: Documenting the implementation of the Matrix model of substance use treatment for opioid users in a South African setting. Addict Behav. 2017;66:132–7. https://doi.org/10.1016/j.addbeh.2016.11.014.
48. Nguyen Huu C, Thuy KC. Application of Matrix model in support of trreatment and recovery for ATS dependents (Nghiên cứu s dụng liệu pháp tâm lý Matrix trong h tr điều trị nghiện và phục hồi chức năng cho ngư i nghiện ma túy t ng h p dạng amphetamine). Hanoi; 2016.
49. Curran GM, Bauer M, Mittman B, Pyne JM, Stetler C. Effectiveness-implementation hybrid designs. Med Care. 2012;50(3):217–26. https://doi.org/10.1097/MLR.0b013e3182408812.
50. Damschroder LJ, Aron DC, Keith RE, Kirsh SR, Alexander JA, Lowery JC. Fostering implementation of health services research findings into practice: a consolidated framework for advancing implementation science. Implement Sci. 2009;4(1):50. https://doi.org/10.1186/1748-5908-4-50.
51. Damschroder LJ, Hagedorn HJ. A guiding framework and approach for implementation research in substance use disorders treatment. Psychol Addict Behav. 2011;25(2):194–205. https://doi.org/10.1037/a0022284.
52. Hanoi Centre for Disease Control. MMT program data. 2021.
53. HCMC Centre for Disease Control. MMT program data. 2021.
54. Roll JM, Shoptaw S. Contingency management: Schedule effects. Psychiatry Res. 2006;144(1):91–3. https://doi.org/10.1016/j.psychres.2005.12.003.
55. Giang LM, Li MJ, Okafor CN, Diep NB, Shoptaw SJ. Correlates of methamphetamine use severity among patients receiving methadone maintenance treatment for opioid use disorder in Vietnam. J Subst Abus Treat. 2022;132:108461. https://doi.org/10.1016/j.jsat.2021.108461.
56. McGregor C, Srisurapanont M, Mitchell A, Longo MC, Cahill S, White JM. Psychometric evaluation of the Amphetamine Cessation Symptom Assessment. J Subst Abus Treat. 2008;34(4):443–9. https://doi.org/10.1016/j.jsat.2007.05.007.
57. Bush K, Kivlahan DR, McDonell MB, Fihn SD, Bradley KA. The AUDIT alcohol consumption questions (AUDIT-C): an effective brief screening test for problem drinking. Ambulatory Care Quality Improvement Project (ACQUIP). Alcohol Use Disorders Identification Test. Arch Intern Med. 1998;158(16):1789–95. https://doi.org/10.1001/archinte.158.16.1789.
58. Clement S, Brohan E, Jeffery D, Henderson C, Hatch SL, Thornicroft G. Development and psychometric properties the Barriers to Access to Care Evaluation scale (BACE) related to people with mental ill health. BMC Psychiatry. 2012;12(1):36. https://doi.org/10.1186/1471-244X-12-36.
59. Lovibond PF, Lovibond SH. The structure of negative emotional states: comparison of the Depression Anxiety Stress Scales (DASS) with the Beck Depression and Anxiety Inventories. Behav Res Ther. 1995;33(3):335–43. https://doi.org/10.1016/0005-7967(94)00075-U.
60. Tran TD, Tran T, Fisher J. Validation of the depression anxiety stress scales (DASS) 21 as a screening instrument for depression and anxiety in a rural community-based cohort of northern Vietnamese women. BMC Psychiatry. 2013;13(1):24. https://doi.org/10.1186/1471-244X-13-24.
61. Sherbourne CD, Stewart AL. The MOS social support survey. Soc Sci Med. 1991;32(6):705–14. https://doi.org/10.1016/0277-9536(91)90150-B.
62. Khuong LQ, Vu T-VT, Huynh V-AN, Thai TT. Psychometric properties of the medical outcomes study: social support survey among methadone maintenance patients in Ho Chi Minh City, Vietnam: a validation study. Subst Abuse Treat Prev Policy. 2018;13(1):8. https://doi.org/10.1186/s13011-018-0147-4.
63. Mai VQ, Sun S, Minh HV, Luo N, Giang KB, Lindholm L, et al. An EQ-5D-5L value set for Vietnam. Qual Life Res Int J Qual Life Asp Treat Care Rehab. 2020;29(7):1923–33. https://doi.org/10.1007/s11136-020-02469-7.
64. UNAIDS. Costing guidelines for HIV prevention strategies. 2000.
65. Hintz J. PASS 12. 2013 [cited 2021 Apr 23]. Available from: https://www.ncss.com/
66. Dai T, Shete S. Time-varying SMART design and data analysis methods for evaluating adaptive intervention effects. BMC Med Res Methodol. 2016;16(1):112. https://doi.org/10.1186/s12874-016-0202-7.
67. Neumann PJ, Sanders GD, Russell LB, Ganiats TG, Siegel JE. Cost-effectiveness in health and medicine: Oxford University Press; 2017. p. 537. https://doi.org/10.1093/acprof:oso/9780190492939.001.0001.
68. Kenward M. The handling of missing data in clinical trials. Clin Investig. 2013;3(3):241–50. https://doi.org/10.4155/cli.13.7.
69. Li P, Stuart EA. Best (but oft-forgotten) practices: missing data methods in randomized controlled nutrition trials. Am J Clin Nutr. 2019;109(3):504–8. https://doi.org/10.1093/ajcn/nqy271.
70. Dawson R, Lavori PW. Design and inference for the intent-to-treat principle using adaptive treatment. Stat Med. 2015;34(9):1441–53. https://doi.org/10.1002/sim.6421.
71. De Crescenzo F, Ciabattini M, D'Alò GL, De Giorgi R, Del Giovane C, Cassar C, et al. Comparative efficacy and acceptability of psychosocial interventions for individuals with cocaine and amphetamine addiction: A systematic review and network meta-analysis. PLoS Med. 2018;15(12):e1002715. https://doi.org/10.1371/journal.pmed.1002715.

Publisher's Note

Springer Nature remains neutral with regard to jurisdictional claims in published maps and institutional affiliations.
